# Violet Arsenic Phosphorus: Switching p-Type into High Performance n-Type Semiconductor by Arsenic Substitution

**DOI:** 10.1007/s40820-025-01956-1

**Published:** 2026-01-12

**Authors:** Rui Zhai, Zhuorui Wen, Xuewen Zhao, Junyi She, Mengyue Gu, Fanqi Bu, Chang Huang, Guodong Meng, Yonghong Cheng, Jinying Zhang

**Affiliations:** 1 State Key Laboratory of Electrical Insulation and Power Equipment, Center of Nanomaterials for Renewable Energy, School of Electrical Engineering, Xi’an Jiaotong University, Xi’an, Shaanxi 710049 People’s Republic of China; 2 State Grid Integrated Energy Service Group Co., Ltd., Beijing, 100052 People’s Republic of China; 3 Instrumental Analysis Center of Xi’an Jiaotong University, Xian Jiaotong University, Xi’an, 710049 People’s Republic of China

**Keywords:** Violet phosphorus, Arsenic substitution, n-type semiconductor, High mobility, Field effect transistor

## Abstract

Violet phosphorus, a recently explored layered elemental semiconductor, has attracted much attention due to its unique photoelectric, mechanical properties, and high hole mobility. Herein, violet arsenic phosphorus has for the first time been synthesized by a molten lead method. The crystal structure of violet arsenic phosphorus (P_83.4_As_0.6_, CSD-2408761) was determined by single crystal X-ray diffraction to have similar structure as that of violet phosphorus, where P12 is occupied by arsenic/phosphorus (As/P) atoms as mixed occupancy sites As1/P12. The arsenic substitution has been demonstrated to tune the band structure of violet phosphorus, switching p-type of violet phosphorus to high-performance n-type violet arsenic phosphorus. The effective electron mass along the < 010 > direction is significantly reduced from 1.792 to 0.515 m_0_ by arsenic substitution, resulting in an extremely high electron mobility of 2622.503 cm^2^ V⁻^1^ s⁻^1^. The field effect transistor built with P_83.4_As_0.6_ nanosheets was measured to have a high electron mobility (137.06 cm^2^ V⁻^1^ s⁻^1^, 61.2 nm), even under ambient conditions for 5 h, much higher than the hole mobility of violet phosphorene nanosheets (4.07 cm^2^ V⁻^1^ s⁻^1^, 73.3 nm). This work provides a new idea for designing phosphorus-based materials for field effect transistors, giving significant potential in complementary metal–oxide–semiconductor applications.

**Supplementary Information:**

The online version contains supplementary material available at 10.1007/s40820-025-01956-1.

## Highlights

- Violet arsenic phosphorus (VP-As) single crystals were synthesized and characterized by single crystal X-ray diffraction to be P_83.4_As_0.6_ (CSD-2408761), the P12 is occupied by arsenic/phosphorus as a mixed occupancy site.
- The p-type VP has been switched into n-type VP-As, the effective electron mass was significantly reduced and resulted in high electron mobility of 2622.503 cm^2^ V^−1^ s^−1^.
- High electron mobility of 137.06 cm^2^ V^−1^ s^−1^ has been achieved from field effect transistor, much higher than the hole mobility of VP.

**Supplementary Information:**

The online version contains supplementary material available at 10.1007/s40820-025-01956-1.

## Graphical abstract

**Figure:**
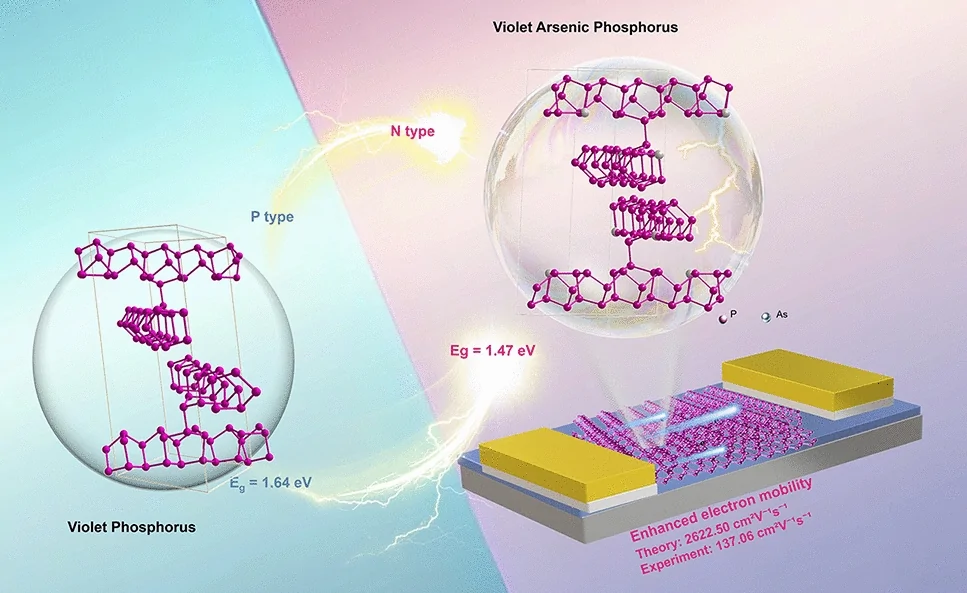

## Introduction

Violet phosphorus (VP) [1], the most stable allotrope of phosphorus, exhibits remarkable performances in anisotropic [2, 3], mechanical [4], electrical [5, 6], optical [7, 8], and photocatalytic [9–12] properties. The effective mass of charge carriers in a semiconductor, which significantly influences their overall semiconductor performance [13, 14], can be modulated through precise alterations in the band structure, leading to substantial improvements in performance for a variety of applications [15–17]. Atomic substitution is a pivotal strategy for bandgap engineering and further modulating the effective mass of charge carriers, tuning the carrier charge mobility of semiconductors. The atomic substitution strategy is widely applied in 2D transition metal dichalcogenides (TMDCs) modification, which involves both the substitution of cationic part or anionic part with highly controllability [18]. For example, the effective charge carrier masses of antimony sulfide (Sb_2_S_3_) were found to be reduced by bismuth substitution of antimony, leading to variations of band dispersion and further enhancing optical properties [19]. The electronic properties of MoS_2_ were modified by substituting molybdenum atoms with niobium, resulting in altered p-type conductivity [20]. The band gap and optical properties of WS_2_ were adjusted by substituting tungsten atoms with niobium, achieving a stable and controllable p-type transport behavior [21]. The bandgap of MoS_2_ was tuned through substituting of sulfur with selenium using selenization [22]. Furthermore, the atomic substitution strategy has also been demonstrated to have potential to modify elemental phosphorus semiconductors. The band gap of black phosphorus (BP) was adjusted from 0.3 to 0.15 eV through the substitution of phosphorus with arsenic, leading to enhancements in its electronic and optical properties [23]. Significant improvements in both environmental stability and hole mobility were also observed in tellurium-substituted BP [24]. However, it is difficult to achieve a controllable atomic substitution of a phosphorus allotrope while maintaining its pristine crystal structure. Nevertheless, it is always a big challenge to grow high-quality single crystals in order to determine their accurate crystal structures.

The accurate crystal structure of semiconductors is crucial for precisely understanding and optimizing material properties [25]. The crystal structure of black phosphorus was determined by single-crystal X-ray diffraction (SC-XRD) in 1965 [26]. However, no single crystal of substituted black phosphorus (black arsenic phosphorus or black antimony phosphorus) was obtained to determine their crystal structures by SC-XRD [23, 27]. The crystal structures of violet phosphorus have been determined by SC-XRD from micro-sized single crystals in 2020 [28]. Afterwards two kinds of antimony-substituted violet phosphorus (P_20.56_Sb_0.44_, P_76_Sb_3.27_) has been synthesized [29, 30]. And the violet P_20.56_Sb_0.44_ has been successfully fabricated to a field effect transistor with 58.96 cm^2^ V^−1^ s^−1^ carrier mobility value (for approximately 30 layers) [29], but the corresponding crystal structure has not been determined by SC-XRD. Besides, although the single crystal structure of violet P_76_Sb_3.27_ was determined by SC-XRD due to the synthesis of large size single crystal blocks [30], the application of violet P_76_Sb_3.27_ in field effect transistor has not been explored. Therefore, the potential of atomic substituted violet phosphorus with accurate single crystal structure for field effect transistors application has not been explored yet, particularly in terms of its potential to alter the effective mass and mobilities of charge carriers.

Herein, we report an arsenic substituted violet phosphorus structure. The violet arsenic phosphorus single crystals has been successfully produced, whose crystal structure has been determined by SC-XRD to have the same crystal structure as that of violet phosphorus. The arsenic substitution has been found to tune the band structure of violet phosphorus, leading to significant modification of effective mass of charge carrier and further altering its charge carrier mobility. The p-type violet phosphorus has been switched into high performance n-type violet arsenic phosphorus by arsenic substitution.

## Experimental Section

### Synthesis of P_83.4_As_0.6_

A mixture of 470 mg amorphous red phosphorus (Aladdin.99.999% metal basis), 284 mg arsenic powder (Aladdin. 99.9% metal basis) and 10 g lead powder (Pb, Ron, 99.99% metal basis) was sealed in a 75 mm long quartz tube with an inner diameter of 14 mm and a wall thickness of 2 mm in a vacuum of 10^–6^ mbar. The quartz tube was then placed horizontally in a muffle furnace. The quartz tube was gradually heated to 630 °C in the muffle furnace for 10 h. The samples were kept at this temperature for 5 h and then cooled to 490 °C at rate of 10 °C/day and then cooled to room temperature naturally. The VP-As (P_83.4_As_0.6_) crystals were then produced inside lead as a grey bump. The VP-As-low (with low content of arsenic) was produced via same process, where 126 mg arsenic powder was adopted.

### Characterization

Transmission electron microscopy (TEM) images, high-resolution TEM (HRTEM) images and SAED patterns were acquired by Lorenz Transmission Electron Microscope (Talos F200X). Scanning electron microscopy (SEM) images were recorded by Quanta 250FEG equipment. X-ray diffraction patterns were obtained from a Bruker D2 PHASER using Cu/Kα radiation (λ = 1.5418 Å) at 40 kV and 30 mA. Raman spectroscopy was taken in a back-scattering geometry using a single monochromator with a microscope (Reinishaw in Via) equipped with CCD array detector and an edge filter. The samples were excited by laser with wavelength of 633 nm. UV–vis-NIR spectrometer (JASCO, V-670) was used to measure the optical features. X-ray photoelectron spectroscopy (XPS) spectra were obtained using a Thermo Fisher ESCALAB spectrometer. Single-crystal X-ray diffraction (SC-XRD) measurements were detected by a Bruker D8 Venture with Mo radiation, PHOTON III High sensitivity two-dimensional detector and Oxford Cryostream 800 plus liquid nitrogen cryogenic system.

### Field Effect Transistor Device Fabrications and Measurements

High quality violet arsenic phosphorus and violt phosphorus crystals were picked up under optical microscope. The P_83.4_As_0.6_ phosphorene or violet phosphorene nanosheets were mechanically exfoliated using blue tape and then transferred onto a SiO_2_/Si substrates (300 nm SiO_2_ as dielectric layer). Subsequently, the substrate was placed on a hot plate at 70 °C for 1 min under ambient conditions, where the tape will lose its adhesion and fall off from exfoliated phosphorene nanosheets. A copper grid mask with four different square pores (length of 52, 73,116, and 155 μm, and grid width of 10 μm) was then fixed on top of the substrate with asistant of hard tape to ensure a tight contact with the substrate. The electrode materials of Ti/Au (10/50 nm) were subsequently deposited on the substrates using an electron beam evaporation system (HHV, Auto-500e) with a deposition rate of 0.5 A s^−1^. A bottom-gated FET based on the exfoliated P_83.4_As_0.6_ phosphorene or violet phosphorene nanosheet as channel material was then obtained fabricated. The electrical properties of the obtained back-gated FET devices were measured under ambient conditions using a Keithley 4200 A Semiconductor Parameter Analyzer.

## Results and Discussion

### Synthesis and Structural Characterizations

**Fig. 1:**
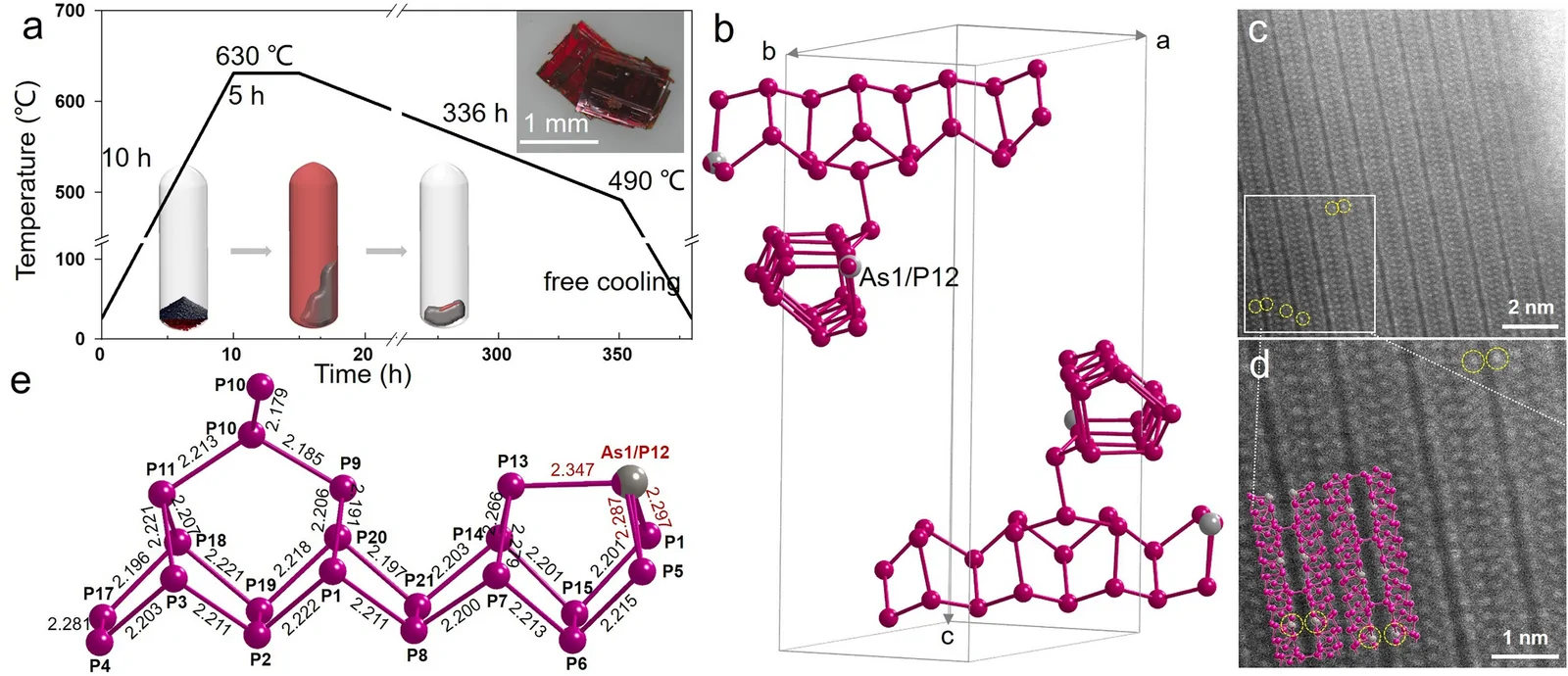
Fabricating process and single crystal structure. a Schematic illustration of violet arsenic phosphorus single crystal growth (inset shows the optical image of violet arsenic phosphorus single crystals after nitric acid treatment). **b** Lattice structure of P_83.4_As_0.6_ single crystal. **c** AC-STEM image of an tilt end of violet arsenic phosphorus. **d** enlarged AC-STEM image with its corresponding structural model (yellow circles represent the occupied positions of arsenic atom). **e** Tubular [P9]P2[P8]P2 substructure and bond lengths of P_83.4_As_0.6_

The arsenic-substituted violet phosphorus single crystals were produced by a modified molten lead method [5] using arsenic powder and amorphous red phosphorus as resources and lead powder as melting agent. Briefly, a mixture of arsenic powder, amorphous red phosphorus, lead powder was sealed in an evacuated quartz tube and then heated in a muffle furnace. The synthesis parameters are illustrated in Fig. 1a. The sample was heated to 630 °C in 10 h, held for 5 h, then gradually cooled down to 490 °C for about 336 h. More experimental details on the synthesis are described in the supporting information. The quartz tube after reaction was removed from the muffle furnace (Fig. S1). Grey lead lumps with violet arsenic phosphorus crystals inside were observed to have gray metallic luster. The violet arsenic phosphorus crystals were then obtained after removing surrounding lead by nitric acid [5] to have a dark red luster (Fig. 1b). The bulk violet arsenic phosphorus was observed to have regular rectangular shapes and flat well-defined facets with a transverse dimension of about 1 mm (inset of Fig. 1a). The layered structure of the bulk violet arsenic phosphorus was also easily visualized from the scanning electron microscopy (SEM) image (Fig. S2).

The lattice structure of violet arsenic phosphorus crystals was then determined by SC-XRD to be monoclinic P_83.4_As_0.6_ with space group of P2/n (CSD-2408761) (Fig. S3). The crystallographic lattice constants were identified as *a* = 9.2075 Å, *b* = 9.1507 Å, *c* = 21.7698 Å, β = 94.388°. Small reflection residual (R_1_) of 7.25% and weighted reflection residual (wR_2_) of 17.69% were obtained from P_83.4_As_0.6_ structure, indicating high fit quality and structural reliability of the SC-XRD. Extinction and absorption corrections were conducted during measurements, where the small amount of missing strong reflections is attributed to the low resolution at very low 2θ angles and slight obstruction for a few data collection points by the beam stop accessory on SC-XRD instrument (Fig. S3). Detailed single-crystal XRD information of the as-produced P_83.4_As_0.6_ is presented in Table S1. A phosphorus atom position (P12) is now occupied by arsenic/phosphorus (As/P) atoms, resulting in the mixed occupancy sites As1/P12, as illustrated in Fig. 1b. The specific coordinates of each phosphorus and arsenic atom in P_83.4_As_0.6_ single crystals are detailed in Table S2. The aberration-corrected scanning transmission electron microscopy (AC-STEM) analysis has been conducted to confirm the occupied position of arsenic atoms. The AC-STEM image is consistent with the structure of violet arsenic phosphorus viewed along < 100 > axis (Fig. 1c&d) in atomic scale. The occupied arsenic atoms were observed to distribute on [P8] cages, well consistent with SC-XRD results.

Some phosphorus atoms of violet phosphorus have been successfully substituted by arsenic to yield violet arsenic phosphorus to keep the similar crystal structure of violet phosphorus [28], where only a slight variation in lattice constants were observed. After atomic replacement, all the As/P-P bond lengths around the mixed occupancy sites As1/P12 (2.347, 2.297, and 2.287 Å respectively with red indication in Fig. 1c) were found to be extended compared with the P-P bond lengths in the original violet phosphorus (2.314, 2.235, and 2.234 Å respectively with red indication in Fig. S4). The single crystal structure of synthesized P_83.4_As_0.6_ from SC-XRD refinement has been further compared with the structure of reported P_20.56_Sb_0.44_ from powder XRD refinement. Atomic substitutional positions in P_83.4_As_0.6_ and P_20.56_Sb_0.44_ was found to both occur at the vertexes of [P8] cages, where the longest P-P bond (2.314 Å) compared to others (2.172 ~ 2.239 Å) in pristine VP was easier to be broken (Fig. S4). The bond length increased from 2.314 Å of VP to 2.347 Å of P_83.4_As_0.6_ after arsenic substitution at one vertex (Fig. 1c), while increased to 2.530 Å for P_20.56_Sb_0.44_ after antimony substitution at two vertexes. The difference is mainly due to the lower heteroatomic substitution rates (0.71% in P_83.4_As_0.6_ and 2.10% in P_20.56_Sb_0.44_), and the more similar atomic size and properties of As-P than Sb-P also leads to smaller internal stresses and relatively weak lattice distortion. Therefore, compared to pristine VP, all of the cell parameters in P_20.56_Sb_0.44_ including *a, b, c* and the overall cell volume were reported to be expanded, while only the b parameter and the overall cell volume in P_83.4_As_0.6_ were demonstrated to be expanded (Table S3).

**Fig. 2:**
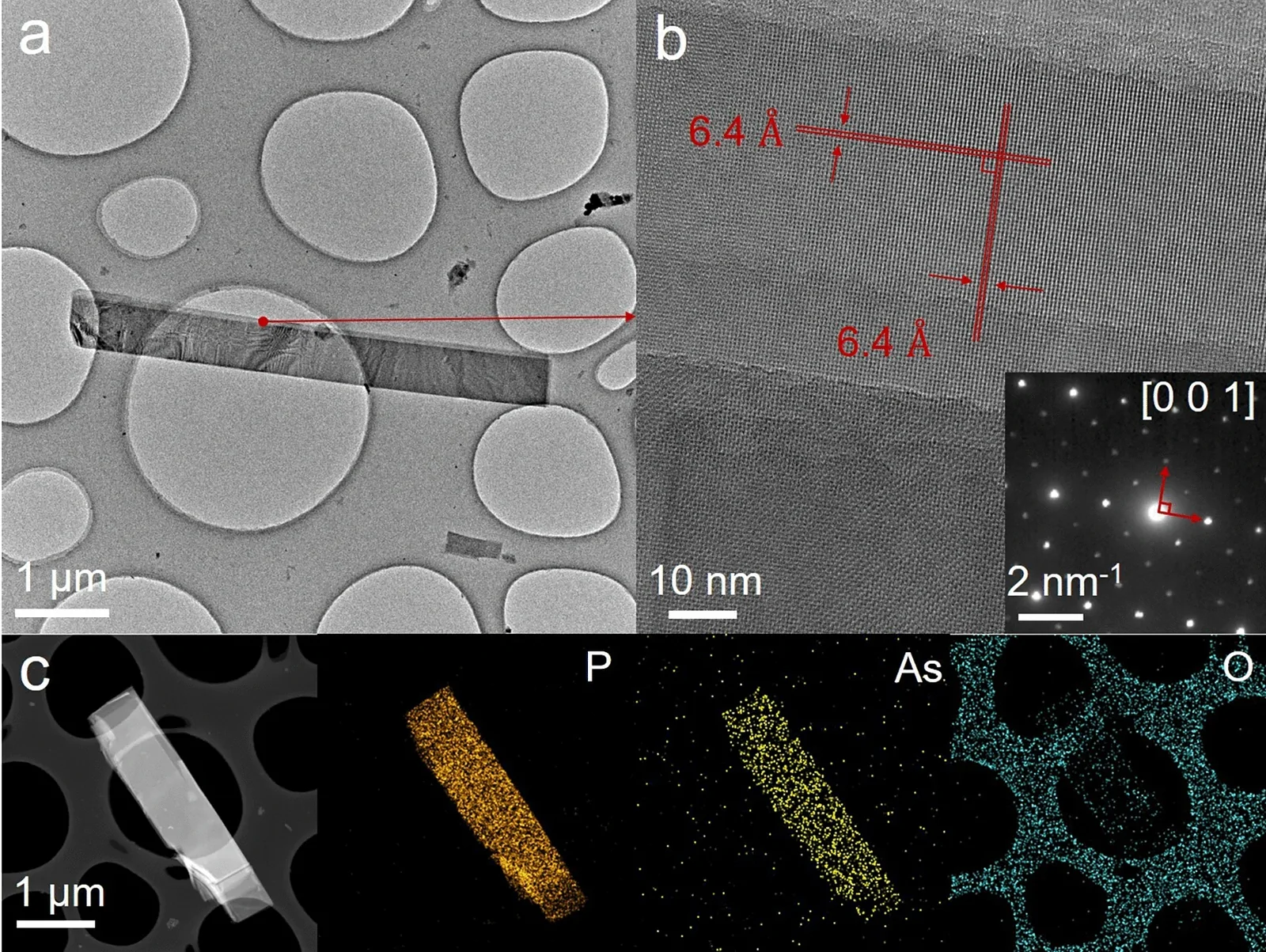
HRTEM images of **a** an exfoliated P_83.4_As_0.6_ phosphorene nanosheet, and **b** the corresponding enlarged structures. The inset shows the SAED pattern with a zone axis of [001]. **c** HAADF image of P_83.4_As_0.6_ phosphorene nanosheet accompanied by corresponding elemental mapping analysis

The P_83.4_As_0.6_ are readily exfoliated into P_83.4_As_0.6_ phosphorene nanosheets using an ultrasonication method. Analogous to violet phosphorene nanosheets [2], the exfoliated P_83.4_As_0.6_ phosphorene nanosheet was observed by transmission electron microscopy (TEM) to display rectangular shapes with erpendicular edges (Fig. 2a). Two sets of crystalline planes, with interplanar spacing of 6.4 Å and perpendicular to each other, were also observed (Fig. 2b). Both the lattice spacing and the intersecting angle are corresponding well with those of the (110) and (1 $\overline{1}$ 0) lattice planes from the crystallographic structure of P_83.4_As_0.6_, as determined by SC-XRD in this study. The inter-planar spacing and angles between planes obtained from the selected area electron diffraction (SAED) pattern with a zone axis of [001] (Fig. 2b, inset) were also found to well consistent with the cell structure determined from the single-crystal XRD of P_83.4_As_0.6_. The P_83.4_As_0.6_ phosphorene nanosheet was also demonstrated by high-angle annular dark field imaging (HAADF, Fig. 2c) and elemental mapping analysis to have a uniform distribution of phosphorus and arsenic elements, with only a negligible amount of oxygen attributed to oxidation on the surface of P_83.4_As_0.6_ phosphorene nanosheet.

**Fig. 3:**
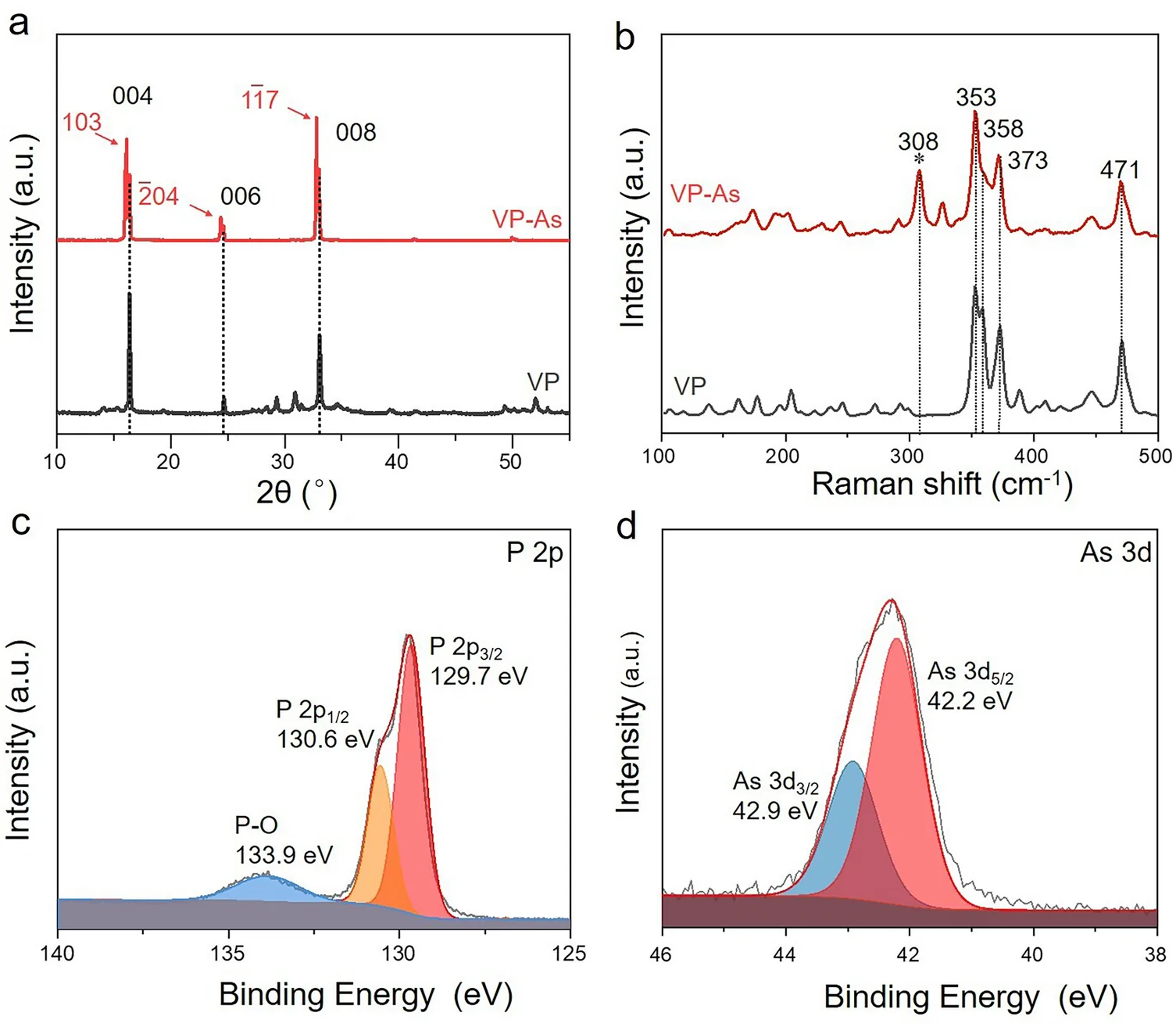
**a** XRD patterns, **b** Raman spectra of P_83.4_As_0.6_ and VP. High-resolution XPS spectra of **c** P2*p* and **d** As 3*d* of P_83.4_As_0.6_

The P_83.4_As_0.6_ structures were further analyzed by powder X-ray diffraction (XRD), Raman scattering and high-resolution X-ray Photoemission Spectroscopy (XPS). Strong peaks with 2θ at 16.3°, 24.6°, and 33.0°, corresponding to the (004), (006), and (008) reflections respectively, were observed in the powder XRD spectrum of mashed P_83.4_As_0.6_ crystals (Fig. 3a, red). Additional diffraction peaks with 2θ at 16.1°, 24.4°, and 32.7°, corresponding to the (103), (-204), and (1 $\overline{1}$ 7) crystal planes of P_83.4_As_0.6_, respectively, were also observable in Fig. 3a (red). No detectable shift was observed from the XRD peaks of mashed P_83.4_As_0.6_ crystals (Fig. 3a, red) compared to those of mashed VP crystals (Fig. 3a, black), confirming the same crystal structures.

The Raman features were measured from P_83.4_As_0.6_ and VP single crystals using a 633 nm excitation laser, as shown in Fig. 3b. The stretching vibrational mode of [P9] cages at 353 cm^−1^ (S^2^_[P9]_), two stretching modes of [P8] at 358 cm^−1^ (S^1^_[P8]_) and 371 cm^−1^ (S^2^_[P8]_) characteristic peaks, and the tangential stretching mode of [P9] along the tubular axis (Tg) at 471 cm^−1^ [28, 31] were observed from both P_83.4_As_0.6_ (Fig. 3b, red) and VP (Fig. 3b, black) single crystals. An additional Raman feature at 308 cm^−1^ were observed from P_83.4_As_0.6_ single crystal (Fig. 3b, red), which is attributed to P–As vibrational mode [32]. The successful substitution of arsenic in P_83.4_As_0.6_ single crystals has been further confirmed by their Raman features.

A weak broad band with binding energy at 133.9 eV corresponding to P-O bond [33] was detected from XPS of P_83.4_As_0.6_ (Fig. 3c), indicating a slightly oxidation of P_83.4_As_0.6_ (Fig. 3c). However, the P-O features are negligible when compared to the prominent peaks of P 2*p*_3/2_ and P 2*p*_1/2_, with binding energies of 129.7 and 130.6 eV, respectively [34]. The binding energies of P 2*p*_3/2_ and P 2*p*_1/2_ from P_83.4_As_0.6_ was found to be slightly lower than those of VP (Fig. S5), attributing to the partial substitution of phosphorus atoms by arsenic atoms where the electron is slightly shifted towards phosphorus in the P-As bonds to increase the nuclear charge experienced by phosphorus [35, 36] The binding energies of As 3*d* [37, 38] were also observed to locate at 42.2 eV (As 3*d*_5/2_) and 42.9 eV (As 3*d*_3/2_) for P_83.4_As_0.6_ (Fig. 3d), significantly higher than that of As metal, further verifying the successful substitution of arsenic atoms (7.2 at% of As) in violet phosphorus.

The quartz tube after reaction is shown in Fig. S6a. The violet arsenic phosphorus crystals (Fig. S6b) were produced inside melted lead (Fig. S6a, yellow box & Fig. S6b) which was easily removed by nitric acid. Some rod-like crystals (Fig. S6a, red box) were found to be with rough spherical tops (Fig. S6c). The top sphere was demonstrated by elemental analysis to be mainly melted lead with slight As, where rods were grown on the melted lead. The presence of phosphorus and arsenic in the smooth rods (Fig. S6c) suggests the early stages of violet arsenic phosphorus nucleation and growth, suggesting the nucleation and crystal growth of violet arsenic phosphorus crystals from spherical lead droplets.

The phosphorus–arsenic rod was observed to have an octagonal intersection (Fig. S6d, f) and layered stacking structure (Fig. S6e) aligned along c-axis of violet arsenic phosphorus (Fig. S6f). Following the growth direction shown in Fig. S6e, the octagonal planes observed in Fig. S6f, g are identified as the ab-planes of the violet arsenic phosphorus crystals. Thus the direction extending from the spherical droplets is determined to be the c-axis direction of the violet arsenic phosphorus crystals, suggesting that violet arsenic phosphorus single crystals nucleate on the surface of the lead droplets and subsequently grow into layered stacks that extend away from the droplet direction. Some uncrystalline irregular lumps (Fig. S6h) were also observed to form around the wall of the quartz tube, which were characterized by EDS to be As–P–Pb composites (Fig. S6i). During the heating process to 630 °C, phosphorus and arsenic are sublimated and subsequently condensed onto the surface of the molten lead, leading to the formation of violet arsenic phosphorus crystals. Upon cooling, the decrease in temperature induces the condensation and nucleation of violet arsenic phosphorus crystals. The nucleation mechanism of violet arsenic phosphorus crystals from lead droplets (Fig. S7) is attributed to the crystal structure of PbP_7_ formed between lead and phosphorus at high temperature (Fig. S7a). The basic skeleton of violet arsenic phosphorus is already existed in PbP_7_ (Fig. S7), where only orange atoms (Fig. S7b) are required to be fluctuated to form [P9]P2[P8]P2 chains (Fig. S7c). Some arsenic atoms with slightly larger atomic radius were fluctuated to replace the orange phosphorus atoms (Fig. S7b) in the relative high strained [P8] cages at high temperature to form violet arsenic phosphorus, consistent well with the crystal structure of violet arsenic phosphorus. Elemental analysis reveals the accumulation of phosphorus and arsenic on the surface of the molten lead droplets, where the nucleation of violet arsenic phosphorus crystals occurs. As the temperature decreases further, the crystals grow along the c-axis, extending away from the lead droplets, with the growth direction being consistent with the c-axis orientation of the violet arsenic phosphorus crystals (Fig. S8). Some arsenic remains within the lead droplets, while the rest co-precipitates with phosphorus to form violet arsenic phosphorus single crystals, thereby explaining the presence of arsenic detected in Fig. S6c. The molten lead method was found to be have better yield and higher crystal quality than other synthesis methods such as chemical vapor transport method (Fig. S9) and direct vapor condensation method (Fig. S10). Most of the as-produced violet arsenic phosphorus products were found to be powders attached to the low-temperature end of quartz tube (Fig. S9b) from the chemical vapor transport method (Fig. S9a), where only a small amount of crystals with up to dozens of micrometres were obtained (Fig. S9c, d). Only amorphous red phosphorus or yellow phosphorus were observed from the direct vapor condensation method (Fig. S10a), which was observed to attached to the quartz tube wall (Fig. S10b). It is hard to found highly crystalline violet arsenic phosphrous from the direct vapor condensation method (Fig. S10c, d). The molten lead method has been demonstrated to be favored for the macro-scale synthesis of high-quality violet arsenic phosphorus crystals, owing to the easy nucleation of violet arsenic phosphorus structure from lead droplets (Fig. S7).

### Electronic Structure Calculations and the Application in Field-Effect Transistors

**Fig. 4:**
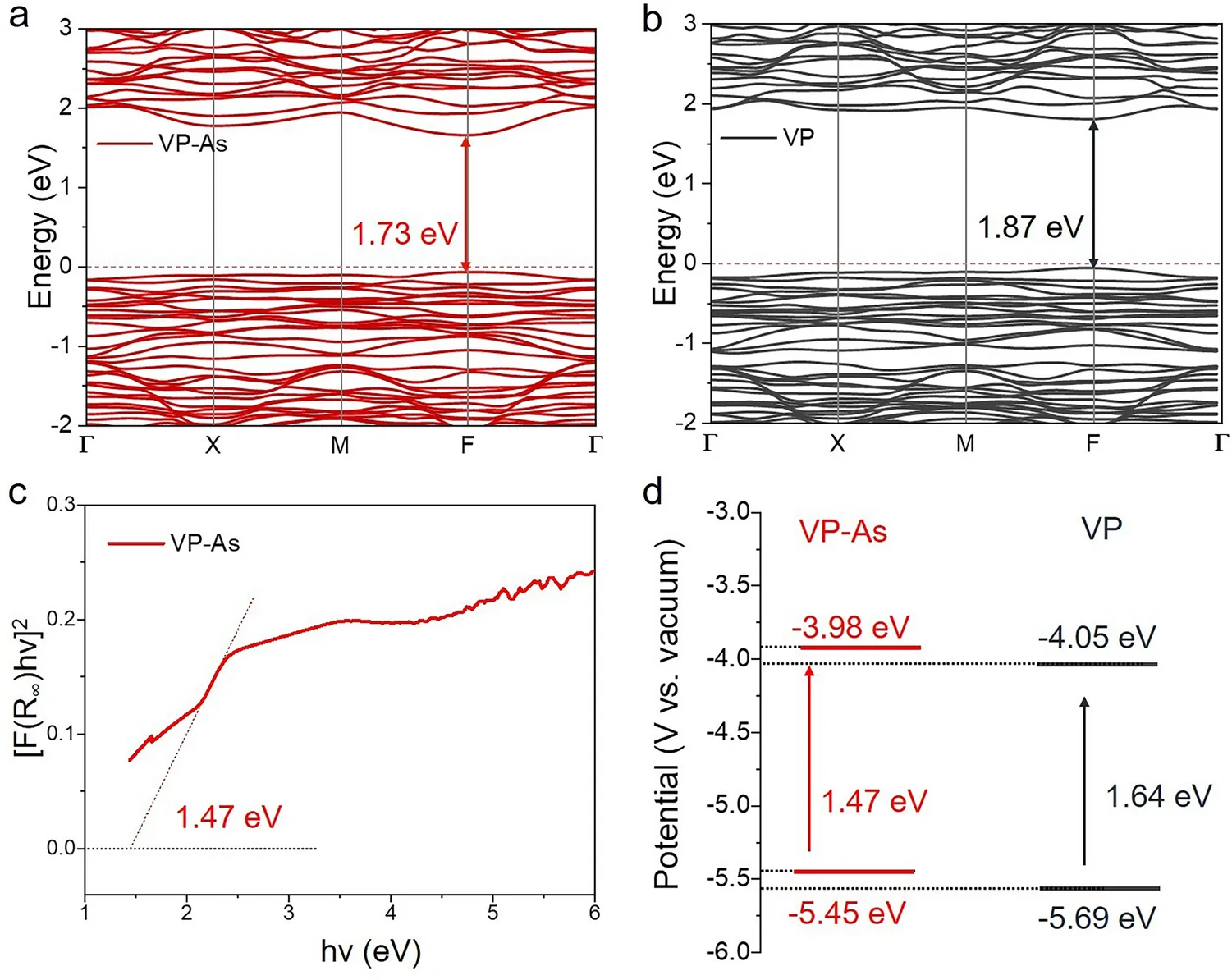
Calculated electronic band structures of **a** P_83.4_As_0.6_ and** b** VP. **c** Diffuse reflectance spectrum of mashed P_83.4_As_0.6_ after Kubelka–Munk treatment. **d** Deduced band edges of P_83.4_As_0.6_ and VP according to vacuum

The electronic structure calculations of VP and P_83.4_As_0.6_ were also performed by the density-functional theory (DFT). Details of calculations are described in the Supporting Information. The energy band structures of P_83.4_As_0.6_ and VP were conducted (Fig. 4a, b). A direct band gap about 1.87 eV was obtained for the VP (two-dimensional section, Fig. 4a), consistent with reported data [28]. The P_83.4_As_0.6_ was predicted to have a direct band gap about 1.73 eV (two-dimensional section, Fig. 4b). The experimental optical band gap (E_g_) of VP and P_83.4_As_0.6_ were determined from Tauc plots using UV/Vis diffuse reflectance spectroscopy (DRS) [39] to be 1.64 eV (Fig. S11c) and 1.47 eV (Fig. 4c), respectively. Additionally, valence band potentials (E_VB, XPS_) of 1.43 eV for VP and 1.19 eV for P_83.4_As_0.6_ were obtained via valence-band X-ray photoelectron spectroscopy (VB-XPS) (Fig. S11b). These values were further adjusted to standard hydrogen electrode levels (E_VB, NHE_) using Eq. (1) [40]:

1 $${\mathrm{E}}_{{{\mathrm{VB}},{\text{ NHE}}}} = \varphi + {\mathrm{E}}_{{{\mathrm{VB}},{\text{ XPS}}}} - {4}.{44}$$

where φ represents the work function of the instrument (4.20 eV), resulting in adjusted values of 1.19 eV for VP and 0.95 eV for P_83.4_As_0.6_.

The valence band potentials relative to vacuum (E_VB, VAC_) were then deduced to be − 5.69 eV for VP and − 5.45 eV for P_83.4_As_0.6_, respectively, according to Eq. (2) [41]:

2 $${\mathrm{E}}_{{{\mathrm{VB}},{\text{ VAC}}}} = - {4}.{5} - {\mathrm{E}}_{{{\mathrm{VB}},{\text{ NHE}}}}$$

Subsequently, the conduction band potentials relative to vacuum (E_CB, VAC_) were deduced to be − 4.05 eV for VP and − 3.98 eV for P_83.4_As_0.6_, using the derived optical band gap E_g_ and E_VB, VAC_ values.

The arsenic substitution of violet phosphorus (P_83.4_As_0.6_) has been demonstrated to significantly modify the band structures of violet phosphorus (Fig. 4). The band gap has been decreased from 1.64 eV of VP to 1.47 eV of P_83.4_As_0.6_ after arsenic substitution, where the conduction band (CB) potential of P_83.4_As_0.6_ (− 3.98 eV) is higher than that of VP (− 4.05 eV) (Fig. 4d). An upshifted conduction band minimum (CBM) is often associated with a reduction in the effective mass of electrons primarily because a higher CBM typically indicates a steeper dispersion curve near the band edge [42, 43]. This steeper curvature results in a larger second derivative of the energy with respect to the wave vector K, which mathematically corresponds to a lower effective mass according to the effective mass tensor formula (3) [44]:

3 $$m^{*} = \hbar ^{2} \left( {d^{2} E/dK^{2} } \right)^{{ - 1}}$$

where ℏ is the reduced Planck constant, and d^2^E/dK^2^ represents the second derivative of the energy with respect to the wave vector, indicating the band’s curvature.

The CBM of P_83.4_As_0.6_ has been demonstrated to be upshifted compared to VP experimentally, resulting in a decreased effective mass and accelerated electron mobility under electric field. The arsenic substation of VP has been demonstrated to effectively enhance the electron mobility and optical absorption, crucial for applications in high-frequency electronics and optoelectronic devices [45].

Detailed theoretical calculations (elastic modulus, deformation potential, effective mass and charge carrier mobility) were conducted to confirm the reductions in effective mass of P_83.4_As_0.6_ phosphorene, aiming to quantitatively assess the effective mass and mobility of charge carriers in both two-dimensional violet phosphrene and P_83.4_As_0.6_ phosphorene (Table 1). The electron mobility of P_83.4_As_0.6_ phosphorene was calculated to increase to 2622.503 cm^2^ V^−1^ s^−1^ along < 010 > direction, which is much higher than that of violet phosphorene (713.640 cm^2^ V^−1^ s^−1^). The substantial reduction in the effective electron mass of P_83.4_As_0.6_ phosphorene along the y-axis (< 010 > , mᵧ*/m₀ = 0.515) facilitates easier and faster electron movement [46, 47], resulting in an enhanced electron mobility. Additionally, the deformation potentials for electrons of P_83.4_As_0.6_ phosphorene (E₁ₓ = 1.161 eV) were predicted to be lower than that of violet phosphorene, suggesting reduced electron scattering [48] and further enhancing its electron mobility. The p-type violet phosphorene has been demonstrated to be switched into high performance n-type violet arsenic phosphorene by arsenic substitution.

**Table 1:** Mobility *μ*_α_, Elastic Modulus *C*_α_, Deformation Potential *E*_1α_, and Effective Mass $m_{\alpha }^{*}$ for violet phosphorene and P_83.4_As_0.6_ phosphorene ^[a]^

	VP		VP-As	
	Electrons	holes	Electrons	holes
$m_{x}^{*} /m_{0}$	0.605	1.305	0.945	1.548
$m_{y}^{*} /m_{0}$	1.792	1.785	0.515	2.561
$E_{1x}$(eV)	1.532	1.599	1.161	1.365
$E_{1y}$(eV)	0.852	0.472	1.204	1.062
$C_{x}^{2D}$(J/m^2^)	46.281	46.281	50.142	50.142
$C_{y}^{2D}$(J/m^2^)	45.882	45.882	50.453	50.453
$\mu_{x}^{2D}$(cm^2^V^−1^s^−1^)	653.768	191.446	859.785	254.203
$\mu_{y}^{2D}$(cm^2^V^−1^s^−1^)	713.640	1592.467	2622.503	143.760

[a] x*:* "the < 100 > direction"; y: "the < 010 > direction"

**Fig. 5:**
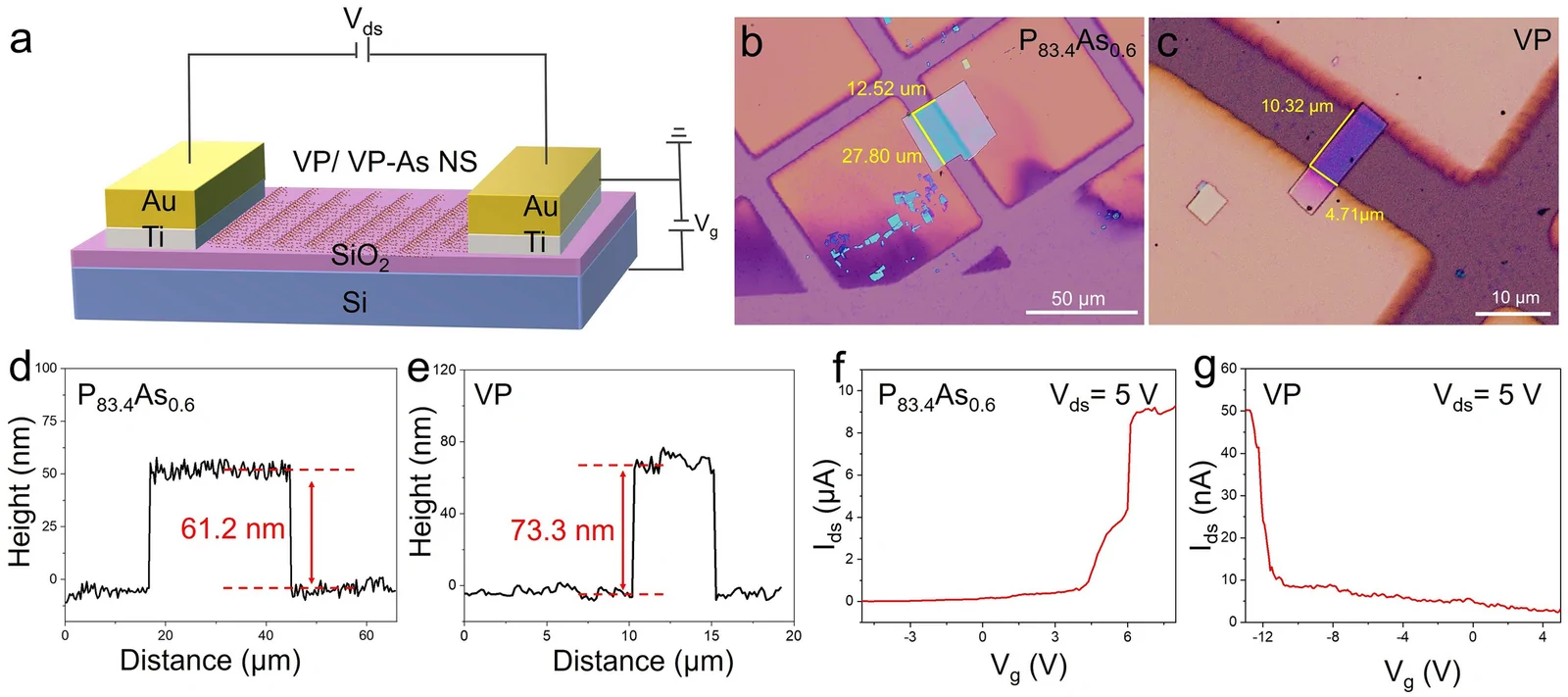
**a** Schematic diagram of a FET based on mechanically exfoliated violet phosphorene/P_83.4_As_0.6_ phosphorene nanosheets. Optical image of a FET based on **b** a P_83.4_As_0.6_ phosphorene nanosheet and **c** a violet phosphorene nanosheet. Height profile of **d** the P_83.4_As_0.6_ phosphorene nanosheet for the device in **b** and **e** the violet phosphorene nanosheet for the device in** c**. Source–drain current as a function of gate voltage obtained from a FET based on **f** a P_83.4_As_0.6_ phosphorene nanosheet (V_ds_ = 5 V) and **g** a violet phosphorene nanosheet (V_ds_ = 5 V)

The Field-Effect Transistor (FET) based on mechanically exfoliated P_83.4_As_0.6_ phosphorene nanosheets was fabricated to characterize its electronic properties. The P_83.4_As_0.6_ phosphorene and violet phosphorene nanosheets were mechanically exfoliated from the as-produced single crystals using a blue tape-assisted mechanical exfoliation technique [5]. A bottom-gated FET based on the exfoliated P_83.4_As_0.6_ phosphorene or violet phosphorene nanosheet as channel material was fabricated on a Si substrate covered with a 300 nm thick thermally oxidized silicon dioxide layer. The source and drain contacts were patterned using a Cu grid mask method [49, 50], followed by the deposition of 10 nm of titanium and 50 nm of gold to establish Au/Ti electrodes. The Ti/Au electrode was found to have better FET performance for violet phosphorene nenosheets than individual gold, silver, or copper electrode, which might be owing to the relative smaller contact resistance (Fig. S11). A schematic diagram of the device structure is illustrated in Fig. 5a. An optical image of the as-fabricated FET based on P_83.4_As_0.6_ phosphorene nanosheet is shown in Fig. 5b, where the conducting channel was measured to be 12.52 μm in length and 27.80 μm in width, respectively. The compared violet phosphorene nanosheet was measured to have a channel of 10.32 μm in length and 4.71 μm in width (Fig. 5c). The thickness of the P_83.4_As_0.6_ phosphorene and violet phosphorene nanosheet was measured by atomic force microscopy (AFM) to be 61.2 nm (Fig. 5d) and 73.3 nm (Fig. 5e), respectively.

The transfer characteristics of the P_83.4_As_0.6_ phosphorene and violet phosphorene nanosheet based FET were assessed by sweeping the gate voltage (V_g_) while applying various drain-source bias voltages (V_ds_) under ambient conditions (Fig. 5f, g). Notably, a characteristic n-type behavior was revealed in the P_83.4_As_0.6_ phosphorene nanosheet-based device. In contrast, a p-type transport behavior was demonstrated by violet phosphorene nanosheet (Fig. 5g), consistent well with reported characteristics of violet phosphorene-based FETs [5, 51]. The p-type semiconductor of violet phosphorene has been confirmed to be switched into n-type P_83.4_As_0.6_ phosphorene by arsenic substitution experimentally. While the antimony substitution one (P_20.56_Sb_0.44_) [29] was found to be still p-type, which might be attributed to the differences in fine electronic structure such as upshifted CBM and altered band gap [52] of P_83.4_As_0.6_. The conversion of p-type violet phosphorene to n-type P_83.4_As_0.6_ phosphorene is important for complementary metal–oxide–semiconductor (CMOS) applications where both p-type and n-type materials are essential, which is regarded as a key manufacturing technology of modern integrated circuit chips [53, 54]. The charge carrier mobility of the P_83.4_As_0.6_ phosphorene-based FETs was calculated using Eq. (4) [55]:

4 $$\mu = \frac{{{\mathrm{d}}I_{{{\mathrm{ds}}}} }}{{{\mathrm{d}}V_{{\mathrm{g}}} }}\frac{{L_{{{\mathrm{ch}}}} }}{{W_{{{\mathrm{ch}}}} C_{{{\mathrm{ox}}}} V_{{{\mathrm{ds}}}} }}$$

The parameter d*I*_ds_/dV_g_, representing the transconductance, was determined from the slope of the transfer characteristic curve. *L*_*c*ℎ_ is the length of the conducting channel, while *W*_*c*ℎ_ represents the channel width. *V*_ds_ refers to the drain-source voltage. The gate capacitance per unit area, *C*_*ox*_, plays a critical role in modulating the device characteristics and was calculated using the formula *C*_*ox*_ = ε_0_ε_*r*_/*d*, where ε_0_ and ε_*r*_ are the dielectric constants of free space and SiO_2_ layer respectively, and *d* is the thickness of the gate dielectric layer.

A high electron mobility of 137.06 cm^2^ V⁻^1^ s⁻^1^ (thickness: 61.2 nm, on/off ratio: 664, subthreshold swing: 114 mV dec^−1^) were deduced from the transfer curve of P_83.4_As_0.6_ phosphorene nanosheet-based FET, which is much higher than the hole mobility (4.07 cm^2^ V⁻^1^ s⁻^1^, thickness: 73.3 nm, on/off ratio: 9.8, subthreshold swing: 130 mV dec^−1^) of violet phosphorene nanosheet-based FET. Furthermore, the substitutional rate of arsenic atoms in violet arsenic phosphorus is crucial for their FET performance. The energy band structures and electron mobility of violet arsenic phosphorus was found to be tuned with different arsenic contents although same crystal structure was adopted (Fig. S12). The Raman features of P-As vibration were detected to increase with increasing arsenic contents for violet arsenic phosphorus (Fig. S12a), whose bandgaps were measured to decrease with increasing arsenic contents (Fig. S12b). The measured valence band maximum (VBM, Fig. S12c) and deduced conducting band minimum (CBM) potions of violet arsenic phosphorus were demonstrated to upshift with increasing arsenic contents (Fig. S12d). The electron mobility of violet arsenic phosphorene nanosheets was also found to increase from 86.38 cm^2^ V⁻^1^ s⁻^1^ for VP-As-low to 137.06 cm^2^ V⁻^1^ s⁻^1^ for VP-As (Fig. S13), where the arsenic content in P_83.4_As_0.6_ was found to approach the highest one where high-quality crystals were able to be obtained from the molten lead method.

The air stability of P_83.4_As_0.6_ phosphorene nanosheet-based FET was found to be much higher than reported phosphorus-based FET, where black phosphorus-based FETs were required to be conducted under inert atmosphere. The P_83.4_As_0.6_ phosphorene nanosheet-based FET were able to be measured under ambient conditions. The performance of P_83.4_As_0.6_ phosphorene nanosheets based FET was demonstrated to be still excellent even after exposure in ambient conditions for 5 h (123.00 cm^2^ V⁻^1^ s⁻^1^, on/off ratio of 558, subthreshold swing of 130 mV dec^−1^) (Fig. S14a, b), while it was detected to be obvisouly deteriorated after exposure for 1 day (61.71 cm^2^ V⁻^1^ s⁻^1^, on/off ratio of 375, subthreshold swing of 176 mV dec^−1^) (Fig. S14c, d). Better performance is expected for P_83.4_As_0.6_ phosphorene nanosheets if encapsulation [45] or heterostructure [56, 57] techniques are adopted.

The carrier mobility of violet phosphorus-based FETs have been significantly enhanced by a small amount of atomic substitution strategy (137.06 cm^2^ V⁻^1^ s⁻^1^ from P_83.4_As_0.6_, 58.96 cm^2^ V^−1^ s^−1^ from P_20.56_Sb_0.44_ [29]), owing to the strong photo-response and significantly reduced effective electron mass by heteroatomic substitution. The FET performance of arsenic substituted violet phosphorus (P_83.4_As_0.6_) was found to represent one of the highest values in phosphorus elemental semiconducting materials (Table S4), especially the unique conversion from p-type to n-type semiconductors which is different from reported elemental phosphorus semiconductor-based FET devices (p-type or holes dominant ambipolar type). The successful conversion of p-type violet phosphorus into high performance n-type violet arsenic phosphorus by small amount of arsenic substitution using a facile one-step synthesis path has been demonstrated to provide significant potential in the modulation of phosphorus-based FET devices compared to the reported functionalization strategy.

The FET performance of P_83.4_As_0.6_ was also compared to reported typical n-type 2D semiconducting materials (Table S5). The electron mobility of P_83.4_As_0.6_ was found to be even higher than most reported n-type 2D semiconducting materials, although the on–off ratio and subthreshold swing of P_83.4_As_0.6_ were found to be moderate due to the rough and simple measurement environments. However, the as-measured values under ambient conditions are still substantially lower than the theoretically predicted electron mobilities of violet phosphorene (713.640 cm^2^ V^−1^ s^−1^) and P_83.4_As_0.6_ phosphorene (2622.503 cm^2^ V^−1^ s^−1^) shown in Table 1. The much lower mobility value obtained from FET is attributed to several factors. One of the biggest influence factors might be the contact resistance due to point contact between phosphorene and electrodes, which is also a common issue between channel materials and the electrodes [58]. This resistance is often caused by several factors including misalignment at the material interfaces, suboptimal contact formations, and potential oxidation at contact points [59–62] And the contact resistance could be further melioratived by the construction of violet arsenic phosphorus-based heterostructures with other 2D materials such as hexagonal boron nitride or graphene, owing to the trap-free interface via perfect van der Waals stacking [63]. Furthermore, the thickness of P_83.4_As_0.6_ phosphorene and violet phosphorene nanosheets were experimentally measured to be 61.2 nm (Fig. 5d) and 73.3 nm (Fig. 5e), which is more than 60 layers. The scattering within the bulk structure is enhanced with increasing thickness, leading to pronounced interactions among charge carriers with its lattice [64, 65] and thereby reducing its charge mobility. Also the electron mobility of P_83.4_As_0.6_ phosphorene and violet phosphorene nanosheets based FET was measured along < 110 > , which is much lower than that along < 010 > . The one-step synthesis path of violet arsenic phosphorus is convenient with relatively lower requirements for equipment compared to reported n-type semiconductors (MoS₂, WS₂, etc.), where the extra lead is easily recycled for following synthesis. The air stability of violet arsenic phosphorus has been significantly enhanced compared to other phosphorus-based semiconductors, although further improvements are still needed. The transition of p-type violet phosphorus into high performance n-type violet arsenic phosphorus by arsenic atomic substitution highlights a significant potential for FETs in electronics, paving the way for advanced transistors suitable for flexible or low-power devices.

Additionally, the appropriate band structure and high carrier mobility characteristics have also found to enable violet arsenic phosphorene nanosheets to exhibit great photocatalytic water splitting properties (545 μmol g^−1^ h^−1^), about 1.8 times of violet phosphorene nanosheets (299 μmol g^−1^ h^−1^) (Fig. S15).

## Conclusions

The violet arsenic phosphorus (P_83.4_As_0.6_) single crystals have been successfully produced, which has been determined by SC-XRD to have the similar crystal structure as that of VP. One phosphorus atom position (P12) is now occupied by arsenic/phosphorus (As/P) atoms, resulting in the mixed occupancy sites As1/P12. The arsenic substitution has been demonstrated to tune the indirect VP into direct P_83.4_As_0.6_ semiconductor. An upshifted conduction band minimum has been relized by arsenic substitution. The effective mass of electrons in the P_83.4_As_0.6_ phosphorene was also found to significantly reduced from 1.792 m_0_ to 0.515 m_0_ along < 010 > direction after arsenic substitution, resulting in a significantly increased electron mobility of 2622.503 cm^2^ V⁻^1^ s⁻^1^ which is much higher than that of violet phosphorene (713.640 cm^2^ V⁻^1^ s⁻^1^). The p-type violet phosphorene has been tuned into high performance n-type violet arsenic phosphorene. A high electron mobility of 137.06 cm^2^V⁻^1^ s⁻^1^ was also obtained from P_83.4_As_0.6_ phosphorene nanosheet (thickness of 61.2 nm) based FET, which is significantly higher than the hole mobility (4.07 cm^2^ V⁻^1^ s⁻^1^) of violet phosphorene nanosheet (73.3 nm thick) based FET under ambient conditions.

## Supplementary Information

Below is the link to the electronic supplementary material.

Supplementary file1 (DOCX 6312 KB)
