# Supplementary material for: Violet Arsenic Phosphorus: Switching p-Type into High Performance n-Type Semiconductor by Arsenic Substitution
Source: Nanomicro Lett. 2026 Jan 12;18:145. doi: 10.1007/s40820-025-01956-1 (PMC12791083; doi:10.1007/s40820-025-01956-1)
Supplement: Supplementary file 1 — Supplementary file1 (DOCX 6312 KB) [file 40820_2025_1956_MOESM1_ESM.docx]

Supporting Information for

**Violet Arsenic Phosphorus: Switching p-Type into High Performance n-Type Semiconductor by Arsenic Substitution**

Rui Zhai^1#^, Zhuorui Wen^1#^, Xuewen Zhao^2#^, Junyi She^1^, Mengyue Gu^1^, Fanqi Bu^1^, Chang Huang^3^, Guodong Meng^1^, Yonghong Cheng^1^, and Jinying Zhang^1^*

^1^ State Key Laboratory of Electrical Insulation and Power Equipment, Center of Nanomaterials for Renewable Energy, School of Electrical Engineering, Xi’an Jiaotong University, Xi’an, Shaanxi 710049, P. R. China

^2^ State Grid Integrated Energy Service Group Co., Ltd., Beijing 100052, P. R. China

^3^ Instrumental Analysis Center of Xi'an Jiaotong University, Xi'an Jiaotong University, Xi'an 710049, P. R. China

^#^Rui Zhai, Zhuorui Wen, and Xuewen Zhao are co-first authors and contributed equally to this work.

*Corresponding author. E-mail: [jinying.zhang@mail.xjtu.edu.cn](mailto:jinying.zhang@mail.xjtu.edu.cn) (Jinying Zhang)

**First-principles Calculations**

Density functional theory (DFT) of bulk VP and P_20.61_As_0.39_ were implemented in the Vienna Ab Initio Simulation Package (VASP) code. The Perdew-Burke-Ernzerhof (PBE) functional within the generalized gradient approximation (GGA) was adopted to describe electronic exchange-correlation energy. The ionic cores were described with the projector augmented wave (PAW) method. The calculated structures were the periodic boundary conditions. A basis set of plane waves was up to an energy cutoff of 500 eV. For the structure optimization and energy calculations, the K-points were set to be 2 × 2 ×2. All of the calculations were continued until the force and energy have converged to less than 0.01 eV Å^-1^ and 10^-4^ eV, respectively. Calculations of violet phosphorene and P_20.61_As_0.39_ phosphorene were carried out on the basis of the framework of DFT and the Vienna ab initio simulation package [S1]. The generalzied gradient approximation proposed by Perdew, Burke, and Ernzerhof is selected for the exchange-correlation potential [S2]. The long range van der Waals interaction is described by the DFT-D3 approach [S3]. The cut-off energy for plane wave is set to 550 eV. The energy criterion is set to 10^−6^ eV in iterative solution of the Kohn-Sham equation. A vacuum layer of 35 Å is added perpendicular to the sheet to avoid artificial interaction between periodic images. The Brillouin zone integration is performed using a 2×2×1 k-mesh. All the structures are relaxed until the residual forces on the atoms have declined to less than 0.01 eV/Å.

Generally, the carrier mobility, μ_2D_, of the 2D semi-conductor, with a view to studying the properties of migration of electrons and holes, can be obtained as follows [S4]:

$$\mu_{2D}=\frac{e\hbar^{3}C_{2D}}{K_{B}Tm_{a}^{*}m_{d}E_{1}^{2}}$$

where e is the electron charge, ℏ is the reduced Planck constant, K_B_ is the Boltzmann constant and T is the temperature (300 K in our calculations). The $m_{a}^{*}$ and $m_{d}=\sqrt{m_{x}^{*}m_{y}^{*}}$ are the effective mass and average effective mass of carriers along the transport direction, respectively. The in-plane stiffness C_2D_ = 2[∂^2^E/∂(Δl/l_0_)^2^]/S_0_, where the E is total energy and S_0_ is the area of the xy plane for the supercell, respectively. The E_1_ = ΔV/(Δl/l_0_) is the deformation potential constant, the ΔV =ΔE_VBM_ or ΔE_CBM_ (strain range from -2% to 2%, calculated using 1% steps), Δl and l_0_ are the deformation of the lattice constants along the transport direction and the intrinsic lattice constants, correspondingly.

**Photocatalytic Hydrogen Evolution Measurements**

10 mg VP-As (P_83.4_As_0.6_) single crystals were first ground followed by the addition of 50 mL of deionized water, which was then exfoliated to phosphorene nanosheets using a Ultrasonic Cell Crusher (JYD-250L) at 25 W for 2 h. About 1 wt.% chloroplatinic acid solution was added to the reaction suspension as precursors to obtain Pt co-catalyst. 0.2 M of ascorbic acid was then added as sacrificial agents. The photocatalytic H_2_ evolution reactions were carried out under irradiation of a Xe lamp (CEL-PF300-T8, 300W, 350 nm - 780 nm) in a quartz cell connected to a glass closed gas circulation and evacuation system (Labsolar-6a, PERFECTLIGHT, Beijing) for 4 h. The production of H_2_ was detected by an online gas chromatography with TCD detector (GC-9790). High pure Argon gas was used as carrier gas.

**Supplementary Figures and Tables**


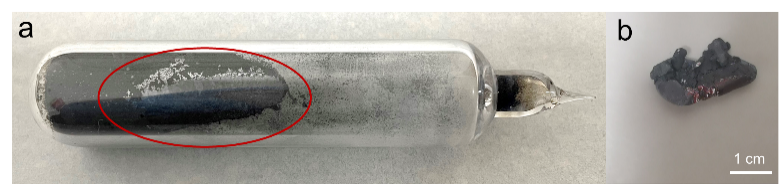


**Fig. S1** Optical images of (**a**) the quartz tube after reaction; (**b**) the as-produced violet arsenic phosphorus wrapped inside molten lead (from red circle of Fig. S1a)


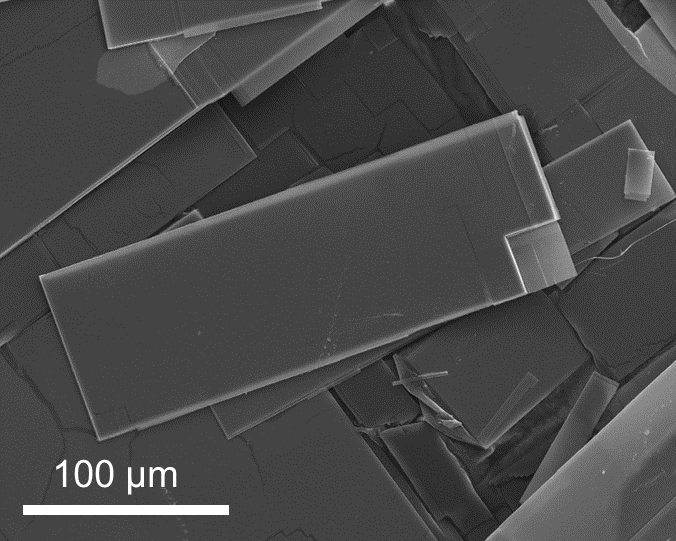


**Fig. S2** SEM image of some pieces of bulk violet arsenic phosphorus


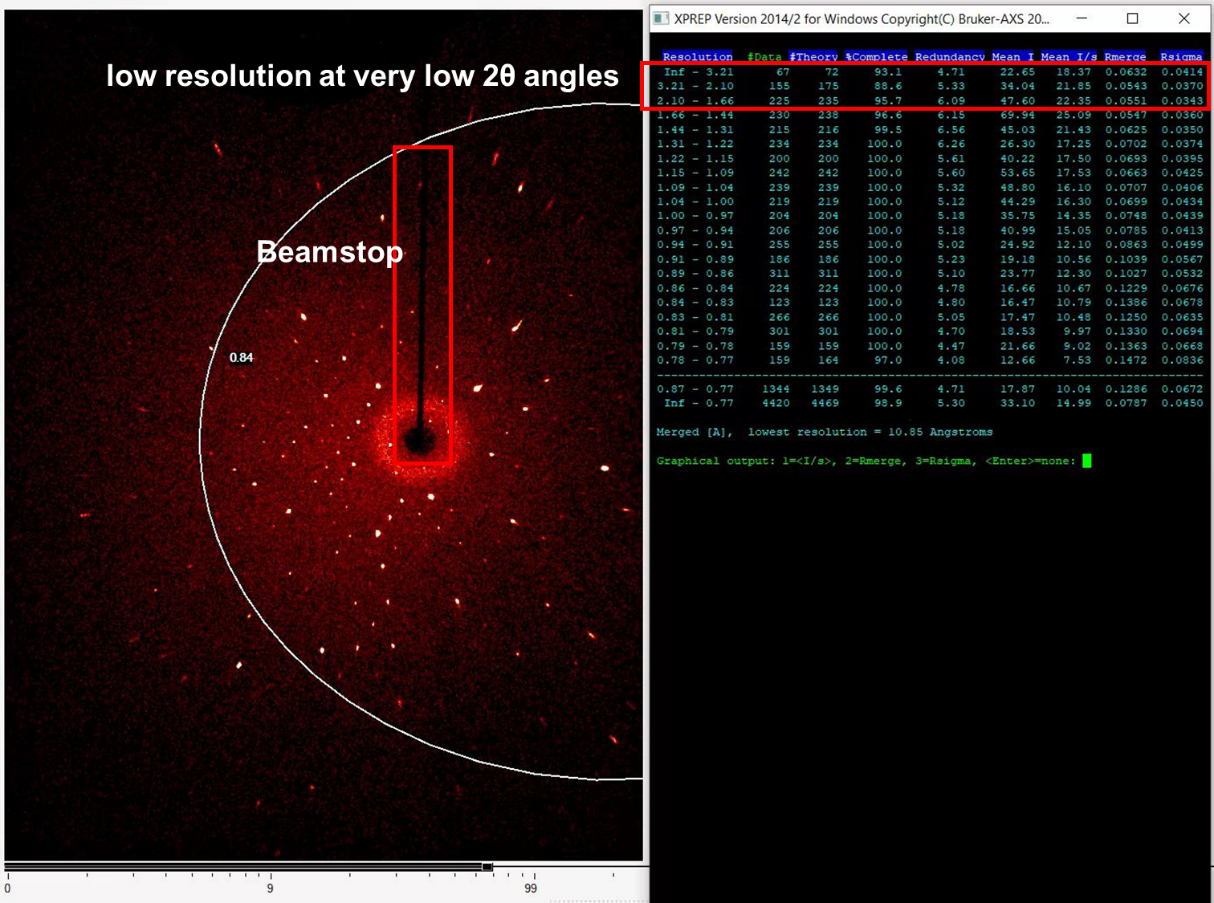


**Fig. S3** Detail picture for the single-crystal X-ray diffraction measurements of P_83.4_As_0.6_


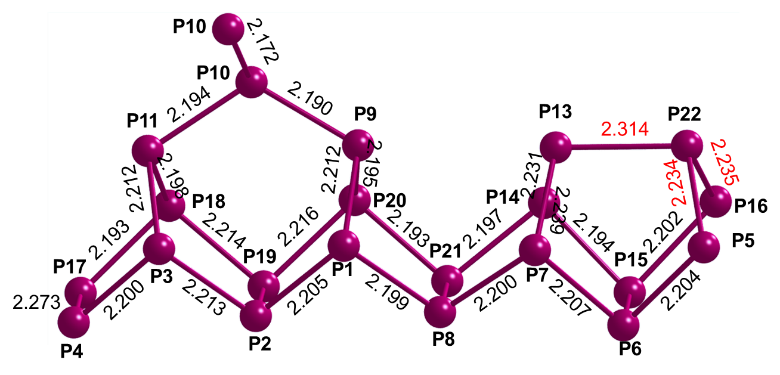


**Fig. S4** Tubular substructure with position number and bond lengths of violet phosphorus


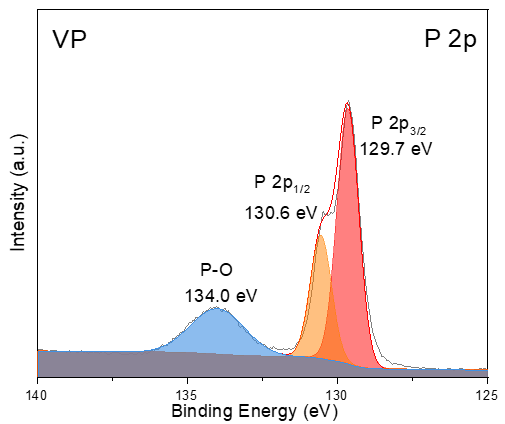


**Fig. S5** High-resolution XPS spectra of P2p of VP


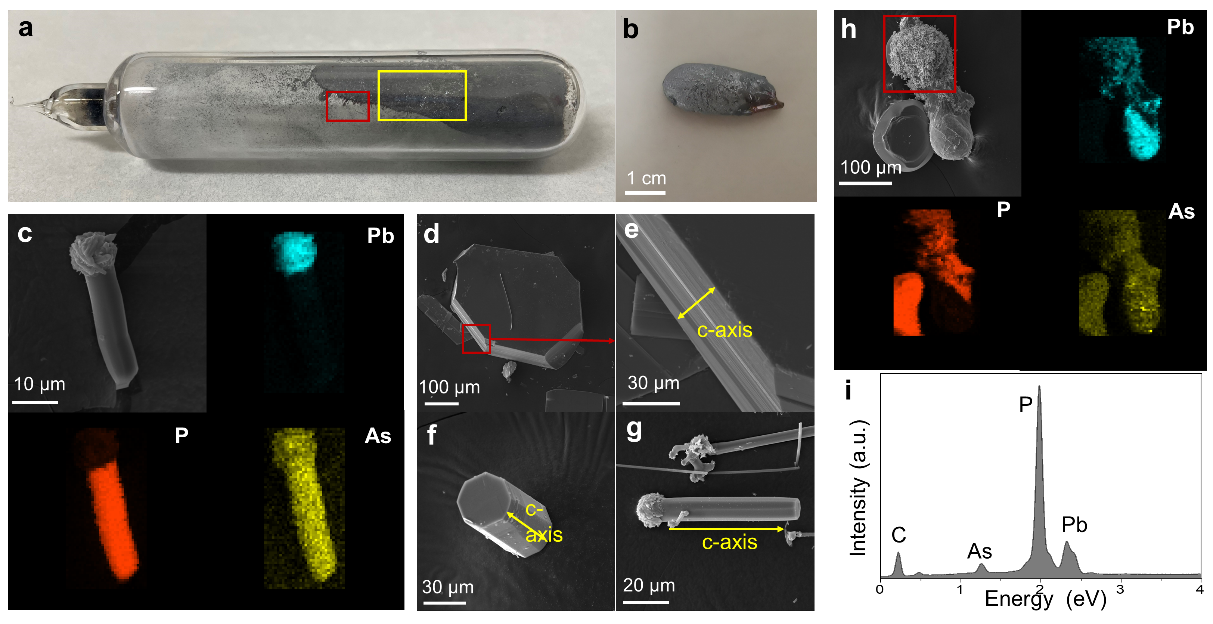


**Fig. S6** Optical images of (**a**) the quartz tube after reaction; (**b**) the as-produced violet arsenic phosphorus wrapped inside molten lead (from yellow box of Figure S3a). (**c**) SEM image of the products from the red box of Figure S3a accompanied by corresponding elemental mapping analysis, SEM image of (**d**) the product from the red box in Figure S3a and (**e**) enlarged layered structure; (**f**)-(**g**) the product from the red box in Figure S3a. (**h**) SEM image accompanied by corresponding elemental mapping analysis and (**i**) EDS spectrum of the As–P–Pb product


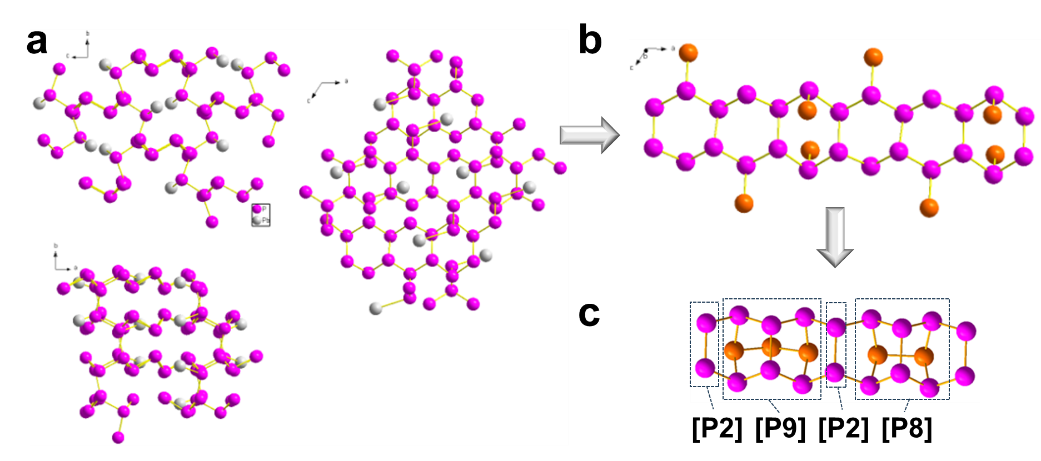


**Fig. S7** The nucleation mechanism of violet arsenic phosphorus crystals from lead droplets. (**a**) Different views of PbP_7_ crystal structure. (**b**) The nearest-neighbor phosphorus structure of PbP_7_ crystals, and (**c**) the corresponding P2[P9]P2[P8] tubular structure of violet phosphorus framework after recombination


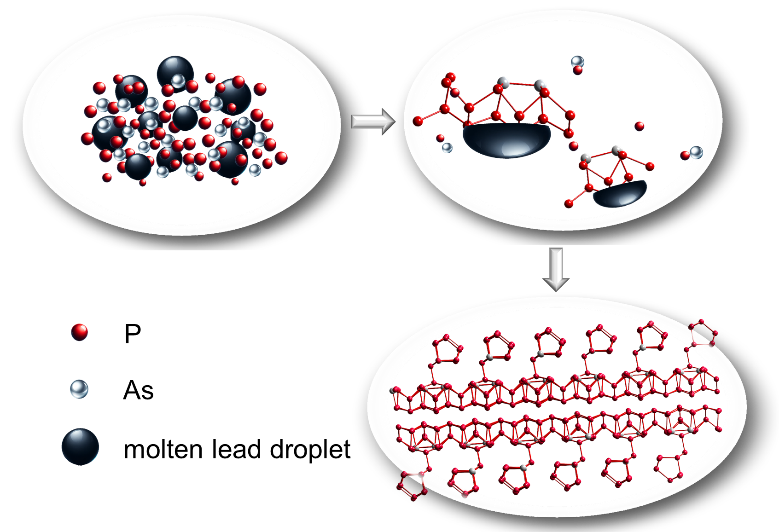


**Fig. S8** Schematic illustration of the growth mechanism of violet arsenic phosphorus crystals


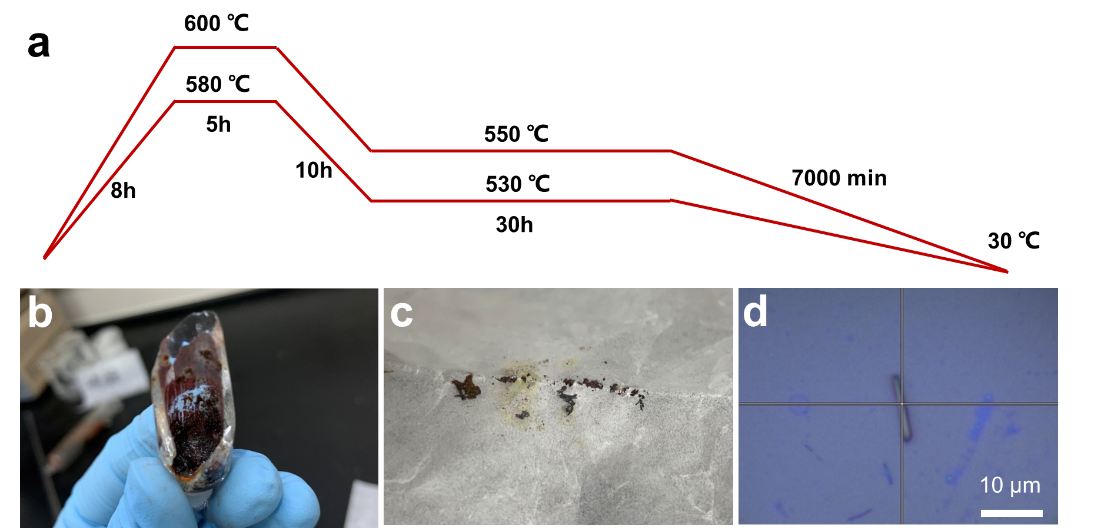


**Fig. S9** (**a**) Synthesis parameters for chemical vapor transport method to produce violet arsenic phosphorus. Optical images of (**b**) the quartz tube after reaction and (**c, d**) the as-produced violet arsenic phosphorus crystals


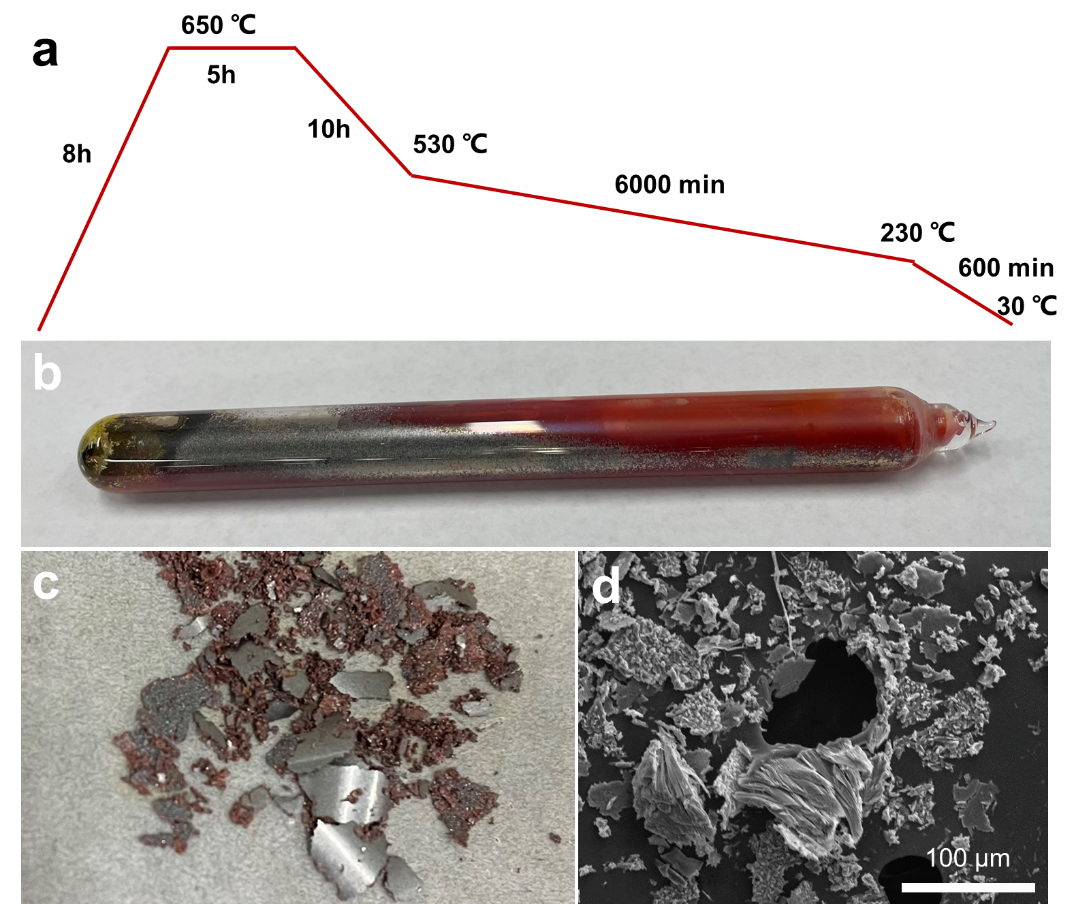


**Fig. S10** (**a**) Synthesis parameters for direct vapor condensation method to produce violet arsenic phosphorus. Optical images of (**b**) the quartz tube after reaction and (**c**) the as-produced violet arsenic phosphorus crystals. (**d**) SEM image of the as-produced violet arsenic phosphorus


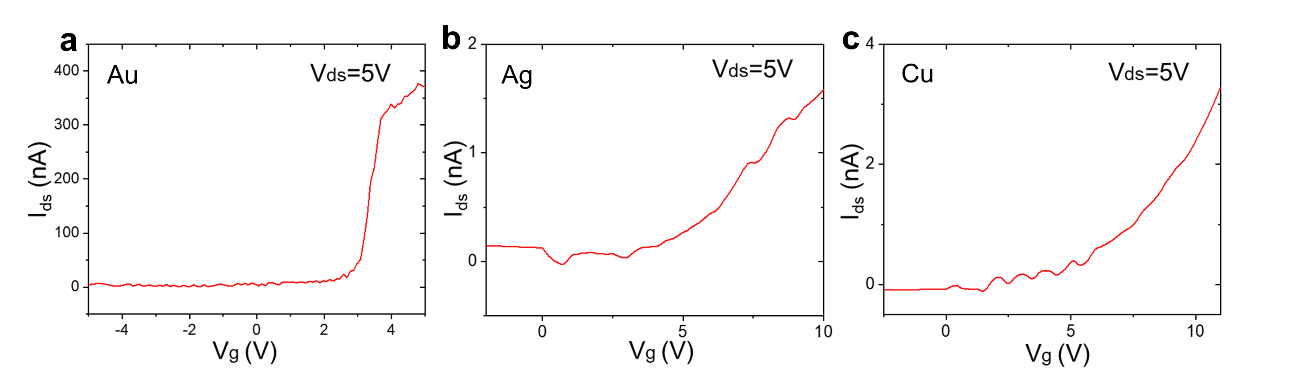


**Fig. S11** The corresponding Source–drain current as a function of gate voltage obtained from a FET based on a VP-As (P_83.4_As_0.6_) nanosheet (V_ds_ = 5 V) with the individual (**a**) gold, (**b**) silver, and (**c**) copper electrode


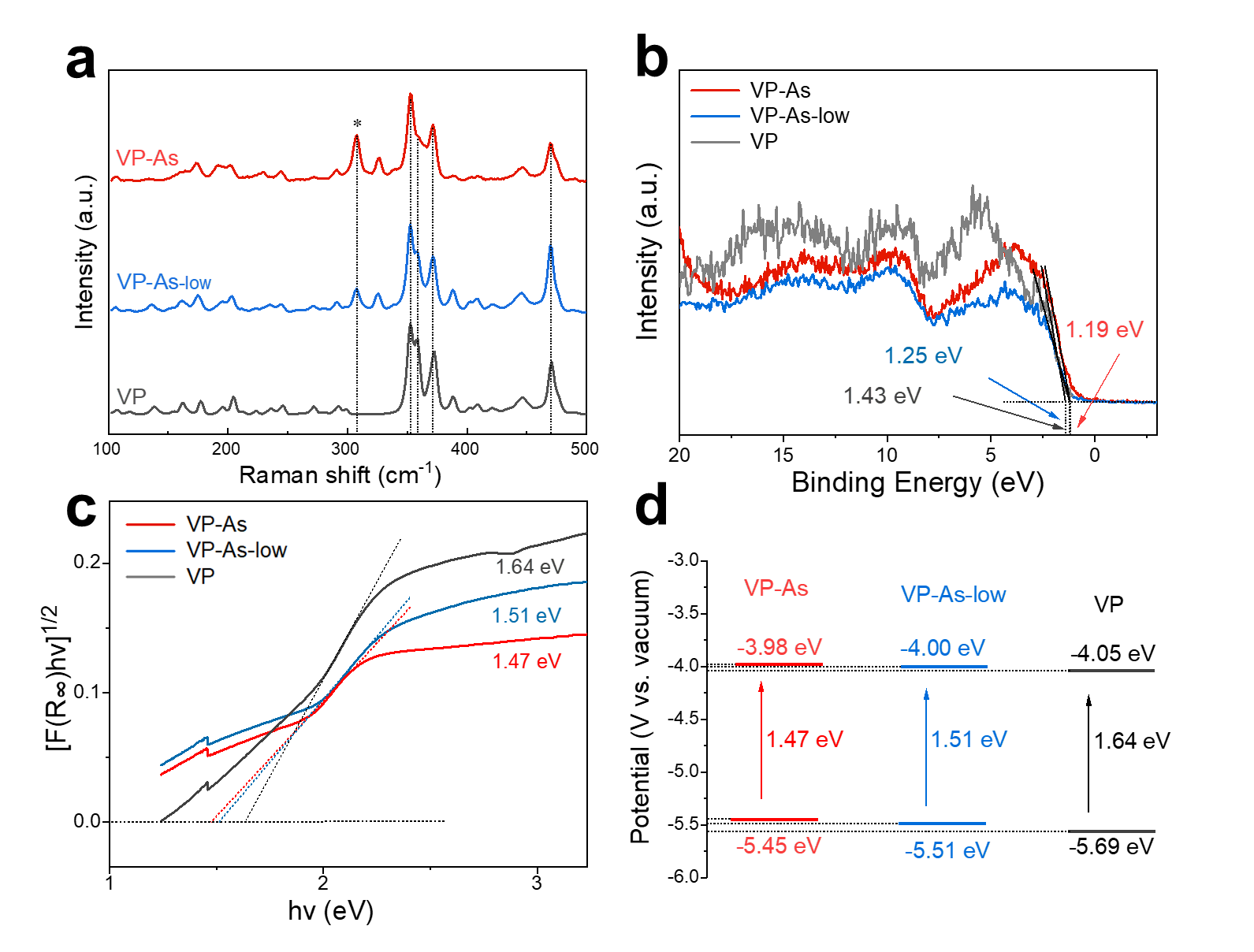


**Fig. S12** (**a**) Raman spectra, (**b**) valence band XPS, (**c**) diffuse reflectance spectra and (**d**) deduced band edges of violet arsenic phosphorus with different substitutional rate of arsenic atoms


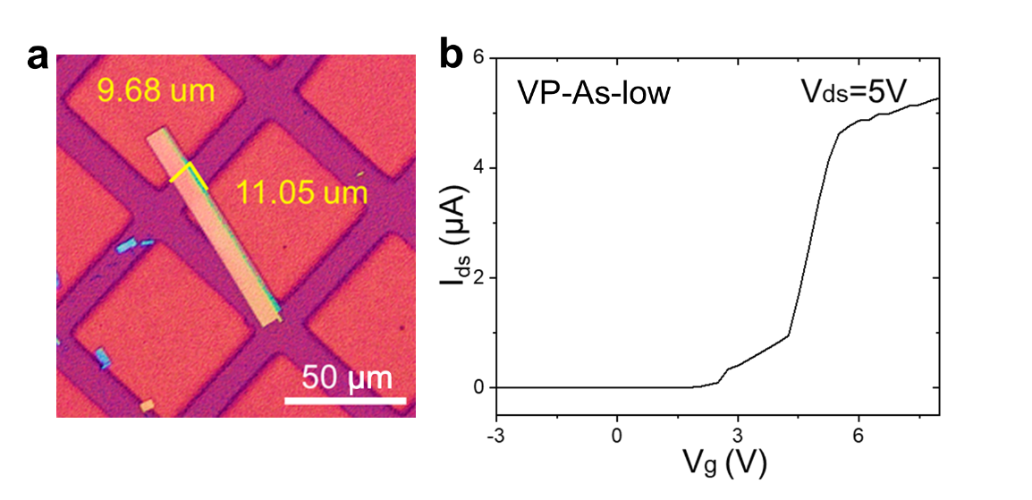


**Fig. S13** (**a**) Optical image and (**b**) corresponding source–drain current (V_ds_ = 5 V) according to gate voltage obtained from a VP-As-low nanosheet based FET


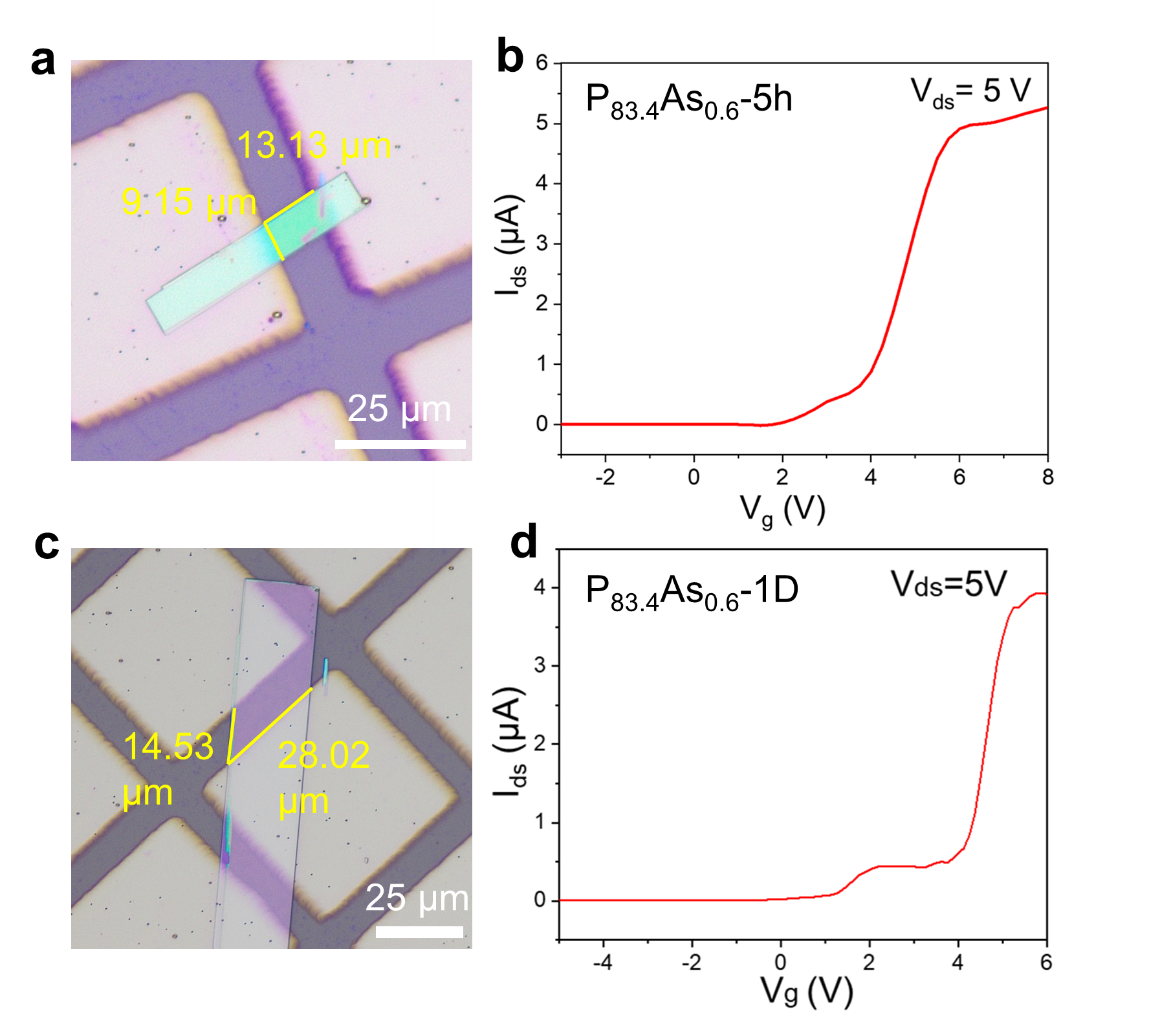


**Fig. S14** (**a**) The optical image and (**b**) corresponding source–drain current (V_ds_ = 5 V) according to gate voltage of a VP-As (P_83.4_As_0.6_) nanosheet based FET after exposure in ambient conditions for 5 hours. (**c**) The optical image and (**d**) corresponding source–drain current (V_ds_ = 5 V) according to gate voltage of a VP-As (P_83.4_As_0.6_) nanosheet based FET after exposure in ambient conditions for 1 day


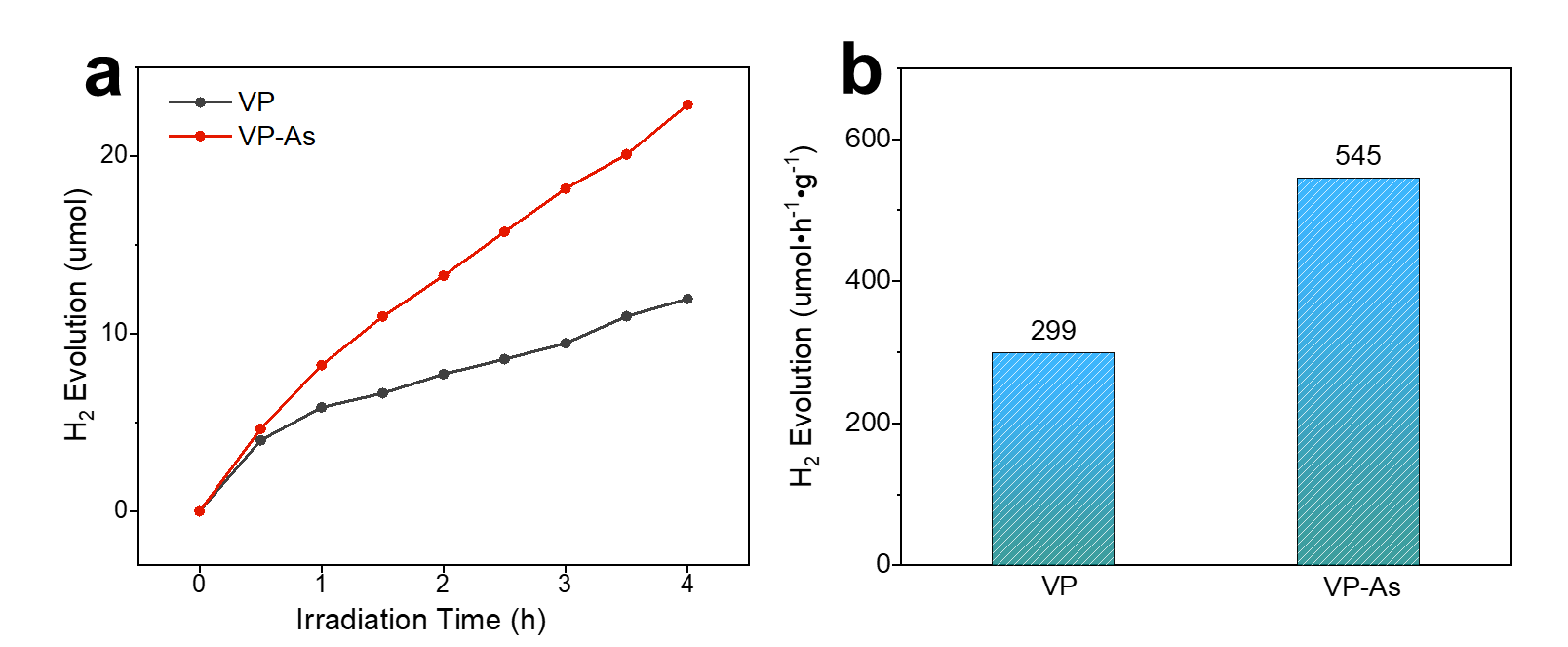


**Fig. S15** (**a**) Photocatalytic H_2_ evolution curves and (**b**) H_2_ evolution rates of violet arsenic phosphorene nanosheets and violet phosphorene nanosheets

**Table S1** Crystal data and structure refinement for a P_83.4_As_0.6_ single crystal

| empirical formula | P_83.4_As_0.6_ |
| --- | --- |
| Formula weight | 2627.85 |
| temperature /K | 302 |
| crystal system | monoclinic |
| space group | P 2/n |
| a /Å | 9.2075(4) |
| b /Å | 9.1507(4) |
| c /Å | 21.7698(10) |
| α/° | 90 |
| β /° | 94.388(2) |
| γ /° | 90 |
| volume /Å^3^ | 1828.84(14) |
| Z | 1 |
| calculated density /g cm^-3^ | 2.386 |
| μ /mm^‑1^ | 2.139 |
| Crystal size/mm^3^ | 0.2 × 0.15 × 0.1 |
| Radiation | MoKα (λ = 0.71073) |
| F(000) | 1271 |
| 2Θ range for data collection /° | 3.752 to 52.742 |
| Index ranges | -11 ≤ h ≤ 11, -11 ≤ k ≤ 11, -28 ≤ l ≤ 28 |
| Reflections collected | 4155 |
| Independent reflections | 3702 |
| Data/restraints/parameters | 3702/12/199 |
| Goodness-of-fit on F^2^ | 1.095 |
| Final R indexes [I>=2σ(I)] | R_1_ = 0.0725, wR_2_ = 0.1730 |
| Final R indexes [all data] | R_1_ = 0.0794, wR_2_ = 0.1769 |
| Largest diff. peak/hole / e Å^-3^ | 2.186/-1.179 |

**Table S2** Atomic coordinates of P_83.4_As_0.6_ crystal unit cell

|  | x | y | z |
| --- | --- | --- | --- |
| P1 | 0.08459 | 0.0732 | 0.68148 |
| P2 | 0.99096 | 0.90461 | 0.61651 |
| P3 | 0.83486 | 0.8191 | 0.67997 |
| P4 | 0.74316 | 0.65214 | 0.61517 |
| P5 | 0.5844 | 0.5518 | 0.67364 |
| P6 | 0.50386 | 0.38688 | 0.60477 |
| P7 | 0.35195 | 0.3222 | 0.67396 |
| P8 | 0.24056 | 0.15201 | 0.61659 |
| P9 | -0.067 | 0.2599 | 0.6722 |
| P10 | -0.27997 | 0.19441 | 0.70065 |
| P11 | 0.64679 | 0.9718 | 0.67338 |
| As1/P12 | 0.391/0.3848 | 0.712/0.7011 | 0.6697/0.6704 |
| P13 | 0.20298 | 0.52139 | 0.67047 |
| P14 | 0.1412 | 0.51267 | 0.56776 |
| P15 | 0.3602 | 0.51717 | 0.53376 |
| P16 | 0.37724 | 0.7447 | 0.56629 |
| P17 | 0.60315 | 0.78161 | 0.54336 |
| P18 | 0.625 | 1.01048 | 0.57304 |
| P19 | -0.14991 | 0.03215 | 0.5441 |
| P20 | -0.12117 | 0.26375 | 0.57244 |
| P21 | 0.10178 | 0.28138 | 0.5438 |

**Table S3** The cell parameters and cell volumes of pristine VP, violet arsenic phosphorus and reported violet antimony phosphorus

|  | Single-crystal XRD data | Powder XRD data | a (Å) | b (Å) | c (Å) | β (°) | V (Å^3^) |
| --- | --- | --- | --- | --- | --- | --- | --- |
| VP | Yes | Yes | 9.210 | 9.128 | 21.893 | 97.7768 | 1823.5(8) |
| VP-As (This work, P_83.4_As_0.6_) | Yes | Yes | 9.2075 | 9.1507 | 21.7698 | 94.388 | 1828.84(14) |
| VP-Sb (P_20.56_Sb_0.44_) | None | Yes | 9.2345 | 9.1732 | 22.6176 | 106.077 | 1841.01(16) |
| VP-Sb (P_76_Sb_3.27_) | Yes | Yes | 9.2146 | 9.1619 | 21.901 | 97.81 | 1831.82 |

**Table S4** Comparison of FET performance of VP-As to reported phosphorus semiconducting materials

| Transistor channel material | Type | Mobility (cm² V⁻¹ s⁻¹) | Refs. |
| --- | --- | --- | --- |
| VP | p | 4.07 | This work |
| VP-As (P_83.4_As_0.6_) | n | 137.06 | This work |
| BP | p | 35 | [S5] |
| BP/HATCN | p | 69 | [S5] |
| BP/HATNA | p | 97 | [S5] |
| exfoliated ultralarge BP with CH_3_COOTBA intercalation | p | 76 | [S6] |
| nonoxidative BP flakes with high crystallinity | p | 60 | [S7] |
| BP-NMP | ambipolar | 50 | [S8] |
| amorphous BP ultrathin film | p | 14 | [S9] |
| BP-C | p | 1995 | [S10] |
| BP | ambipolar | 38 (electrons),  172 (holes) | [S11] |
| BP | p | 100 | [S12] |

**Table S5** Comparison of FET performance of VP-As to reported n-type 2D semiconducting materials

| Transistor channel material | Mobility (cm² V⁻¹ s⁻¹) | on/off ratio | subthreshold swing (mV/dec) | Refs. |
| --- | --- | --- | --- | --- |
| VP-As (P_83.4_As_0.6_) | 137.06 | 664 | 114 | This work |
| multilayer (ML) MoS_2_ islands | 70 | 10^8^ | 150 | [S13] |
| MoS_2_ | 30 | Not mentioned | Not mentioned | [S14] |
| MoS_2_ under tensile strain | 185 | Not mentioned | Not mentioned | [S15] |
| metal-agglomeration-suppressed MoS_2_ films | 59.8 | 10^5^ | 500 | [S16] |
| MoS_2_-PPh_3_ | 60 | Not mentioned | Not mentioned | [S17] |
| MoS_2_-ReO_3_ | 46.3 | Not mentioned | Not mentioned | [S18] |
| MoS_2_-O_2_ | 100 | 10^9^ | 80 | [S19] |
| MoS_2_-NMP | 22.2 | 10^5^ | Not mentioned | [S20] |
| MoS_2_-DMSO | 29.2 | Not mentioned | Not mentioned | [S20] |
| MoS_2_-DMF | 26.6 | Not mentioned | Not mentioned | [S20] |
| WS_2_ | 33 | Not mentioned | Not mentioned | [S14] |
| WS_2_ | 140 | 10^6^ | Not mentioned | [S21] |
| WS_2_ encapsulated in hexagonal BN | 100 | 10^5^ | Not mentioned | [S22] |
| monolayered triangular WS_2_ flakes | 50.5 | 10^7^ | Not mentioned | [S23] |
| WS_2_-Cl | 60 | 4×10^6^ | Not mentioned | [S24] |
| MoSe_2_ | 59.4 | Not mentioned | Not mentioned | [S25] |
| self-assembled monolayers modified MoSe_2_ | 166.5 | Not mentioned | Not mentioned | [S25] |
| bilayer MoSe_2_ | 65 | 10^5^ | Not mentioned | [S26] |
| WSe_2_ with amorphous BN capping layer | 7.42 | 10^6^ | Not mentioned | [S27] |
| WSe_2_-DETA | 25 | Not mentioned | Not mentioned | [S28] |
| WSe_2_-S | 68.2 | Not mentioned | Not mentioned | [S29] |
| MoTe_2_ | ≈ 43 | 10^6^ | Not mentioned | [S30] |

**Supplementary References**

1. G. Kresse, D. Joubert, From ultrasoft pseudopotentials to the projector augmented-wave method. Phys. Rev. B **59**(3), 1758–1775 (1999). <https://doi.org/10.1103/physrevb.59.1758>
2. J. Perdew, K. Burke, M. Ernzerhof, Generalized gradient approximation made simple. Phys. Rev. Lett. **77**(18), 3865–3868 (1996). <https://doi.org/10.1103/PhysRevLett.77.3865>
3. S. Grimme, J. Antony, S. Ehrlich, H. Krieg, A consistent and accurate *ab initio* parametrization of density functional dispersion correction (DFT-D) for the 94 elements H-Pu. J. Chem. Phys. **132**(15), 154104 (2010). <https://doi.org/10.1063/1.3382344>
4. J. Qiao, X. Kong, Z.-X. Hu, F. Yang, W. Ji, High-mobility transport anisotropy and linear dichroism in few-layer black phosphorus. Nat. Commun. **5**, 4475 (2014). <https://doi.org/10.1038/ncomms5475>
5. H. Shi, S. Fu, Y. Liu, C. Neumann, M. Wang et al., Molecularly engineered black phosphorus heterostructures with improved ambient stability and enhanced charge carrier mobility. Adv. Mater. **33**(48), 2105694 (2021). <https://doi.org/10.1002/adma.202105694>
6. N. Wang, N. Mao, Z. Wang, X. Yang, X. Zhou et al., Electrochemical delamination of ultralarge few-layer black phosphorus with a hydrogen-free intercalation mechanism. Adv. Mater. **33**(1), e2005815 (2021). <https://doi.org/10.1002/adma.202005815>
7. J. Li, C. Chen, S. Liu, J. Lu, W.P. Goh et al., Ultrafast electrochemical expansion of black phosphorus toward high-yield synthesis of few-layer phosphorene. Chem. Mater. **30**(8), 2742–2749 (2018). <https://doi.org/10.1021/acs.chemmater.8b00521>
8. J. Kang, J.D. Wood, S.A. Wells, J.-H. Lee, X. Liu et al., Solvent exfoliation of electronic-grade, two-dimensional black phosphorus. ACS Nano **9**(4), 3596–3604 (2015). <https://doi.org/10.1021/acsnano.5b01143>
9. Z. Yang, J. Hao, S. Yuan, S. Lin, H.M. Yau et al., Field-effect transistors based on amorphous black phosphorus ultrathin films by pulsed laser deposition. Adv. Mater. **27**(25), 3748–3754 (2015). <https://doi.org/10.1002/adma.201500990>
10. W.C. Tan, Y. Cai, R.J. Ng, L. Huang, X. Feng et al., Few-layer black phosphorus carbide field-effect transistor *via* carbon doping. Adv. Mater. **29**(24), 1700503 (2017). <https://doi.org/10.1002/adma.201700503>
11. S. Das, M. Demarteau, A. Roelofs, Ambipolar phosphorene field effect transistor. ACS Nano **8**(11), 11730–11738 (2014). <https://doi.org/10.1021/nn505868h>
12. A. Kumar, K. Intonti, L. Viscardi, O. Durante, A. Pelella et al., Memory effect and coexistence of negative and positive photoconductivity in black phosphorus field effect transistor for neuromorphic vision sensors. Mater. Horiz. **11**(10), 2397–2405 (2024). <https://doi.org/10.1039/d4mh00027g>
13. H. Xu, H. Zhang, Z. Guo, Y. Shan, S. Wu et al., High-performance wafer-scale MoS_2_ transistors toward practical application. Small **14**(48), e1803465 (2018). <https://doi.org/10.1002/smll.201803465>
14. A. Sebastian, R. Pendurthi, T.H. Choudhury, J.M. Redwing, S. Das, Benchmarking monolayer MoS_2_ and WS(2) field-effect transistors. Nat. Commun. **12**(1), 693 (2021). <https://doi.org/10.1038/s41467-020-20732-w>
15. X. Liu, B. Erbas, A. Conde-Rubio, N. Rivano, Z. Wang et al., Deterministic grayscale nanotopography to engineer mobilities in strained MoS_2_ FETs. Nat. Commun. **15**, 6934 (2024). <https://doi.org/10.1038/s41467-024-51165-4>
16. K.H. Jung, S.J. Yun, Y. Choi, J.H. Cho, J.W. Lim et al., Metal-agglomeration-suppressed growth of MoS_2_ and MoSe_2_ films with small sulfur and selenium molecules for high mobility field effect transistor applications. Nanoscale **10**(32), 15213–15221 (2018). <https://doi.org/10.1039/C8NR03778G>
17. D. Lee, J.J. Lee, Y.S. Kim, Y.H. Kim, J.C. Kim et al., Remote modulation doping in van der Waals heterostructure transistors. Nat. Electron. **4**(9), 664–670 (2021). <https://doi.org/10.1038/s41928-021-00641-6>
18. X. Ma, J. Zhang, J. Lai, M. Zhang, J. Zheng et al., Gradient rhenium doping enabled tunable anisotropic valleytronic material based on monolayer molybdenum disulfide. 2D Mater. **8**(3), 035031 (2021). <https://doi.org/10.1088/2053-1583/abf762>
19. J. Tang, Z. Wei, Q. Wang, Y. Wang, B. Han et al., *In situ* oxygen doping of monolayer MoS_2_ for novel electronics. Small **16**(42), 2004276 (2020). <https://doi.org/10.1002/smll.202004276>
20. X.-K. Li, R.-X. Sun, H.-W. Guo, B.-W. Su, D.-K. Li et al., Controllable doping of transition-metal dichalcogenides by organic solvents. Adv. Electron. Mater. **6**(3), 1901230 (2020). <https://doi.org/10.1002/aelm.201901230>
21. D. Ovchinnikov, A. Allain, Y.-S. Huang, D. Dumcenco, A. Kis, Electrical transport properties of single-layer WS_2_. ACS Nano **8**(8), 8174–8181 (2014). <https://doi.org/10.1021/nn502362b>
22. Y. Wang, T. Sohier, K. Watanabe, T. Taniguchi, M.J. Verstraete et al., Electron mobility in monolayer WS_2_ encapsulated in hexagonal boron-nitride. Appl. Phys. Lett. **118**(10), 102105 (2021). <https://doi.org/10.1063/5.0039766>
23. Y. Yue, J. Chen, Y. Zhang, S. Ding, F. Zhao et al., Two-dimensional high-quality monolayered triangular WS_2_ flakes for field-effect transistors. ACS Appl. Mater. Interfaces **10**(26), 22435–22444 (2018). <https://doi.org/10.1021/acsami.8b05885>
24. L. Yang, K. Majumdar, H. Liu, Y. Du, H. Wu et al., Chloride molecular doping technique on 2D materials: WS_2_ and MoS_2_. Nano Lett. **14**(11), 6275–6280 (2014). <https://doi.org/10.1021/nl502603d>
25. K. Zhao, D. He, X. Liu, F. Ren, J. Wang et al., Enhance carrier diffusion of monolayer MoSe_2_ by interface engineering. ACS Appl. Mater. Interfaces **16**(26), 34349–34357 (2024). <https://doi.org/10.1021/acsami.4c05143>
26. Y. Li, K. Zhang, F. Wang, Y. Feng, Y. Li et al., Scalable synthesis of highly crystalline MoSe_2_ and its ambipolar behavior. ACS Appl. Mater. Interfaces **9**(41), 36009–36016 (2017). <https://doi.org/10.1021/acsami.7b10693>
27. Z. Lu, M. Zhu, G. Zhang, W. Liu, S. Han et al., Electrical characteristics of WSe_2_ transistor with amorphous BN capping layer. Results Phys. **38**, 105568 (2022). <https://doi.org/10.1016/j.rinp.2022.105568>
28. H.G. Ji, P. Solís-Fernández, D. Yoshimura, M. Maruyama, T. Endo et al., Chemically tuned p- and n-type WSe_2_ monolayers with high carrier mobility for advanced electronics. Adv. Mater. **31**(42), 1903613 (2019). <https://doi.org/10.1002/adma.201903613>
29. X. Duan, C. Wang, Z. Fan, G. Hao, L. Kou et al., Synthesis of WS_2_*_x_*Se_2–2_*_x_* alloy nanosheets with composition-tunable electronic properties. Nano Lett. **16**(1), 264–269 (2016). <https://doi.org/10.1021/acs.nanolett.5b03662>
30. S. Cho, S. Kim, J.H. Kim, J. Zhao, J. Seok et al., DEVICE TECHNOLOGY. Phase patterning for ohmic homojunction contact in MoTe₂. Science **349**(6248), 625–628 (2015). <https://doi.org/10.1126/science.aab3175>
